# New Perspectives on Diagnosis and Therapy of Malignant Pleural Mesothelioma

**DOI:** 10.3389/fonc.2018.00091

**Published:** 2018-04-03

**Authors:** Marika Rossini, Paola Rizzo, Ilaria Bononi, Anthony Clementz, Roberto Ferrari, Fernanda Martini, Mauro G. Tognon

**Affiliations:** ^1^Department of Morphology, Surgery and Experimental Medicine, Section of Pathology, Oncology and Experimental Biology, School of Medicine, University of Ferrara, Ferrara, Italy; ^2^Department of Natural Sciences and Geography, Concordia University Chicago, River Forest, IL, United States; ^3^Department of Medical Sciences, Section of Internal Medicine and Cardiorespiratory, School of Medicine, University of Ferrara, Ferrara, Italy; ^4^E.S. Health Science Foundation, GVM Care & Research, Maria Cecilia Hospital, Cotignola, Italy

**Keywords:** malignant pleural mesothelioma, asbestos, gene, biomarker, microRNA, simian virus 40, Notch, therapy

## Abstract

Malignant pleural mesothelioma (MPM) is a rare, but severe form of cancer, with an incidence that varies significantly within and among different countries around the world. It develops in about one to two persons per million of the general population, leading to thousands of deaths every year worldwide. To date, the MPM is mostly associated with occupational asbestos exposure. Asbestos represents the predominant etiological factor, with approximately 70% of cases of MPM with well-documented occupational exposure to asbestos, with the exposure time, on average greater than 40 years. Environmental exposure to asbestos is increasingly becoming recognized as a cause of mesothelioma, together with gene mutations. The possible roles of other cofactors, such as viral infection and radiation exposure, are still debated. MPM is a fatal tumor. This cancer arises during its early phase without clinical signs. Consequently, its diagnosis occurs at advanced stages. Standard clinical therapeutic approaches include surgery, chemo- and radiotherapies. Preclinical and clinical researches are making great strides in the field of this deadly disease, identifying new biomarkers and innovative therapeutic approaches. Among the newly identified markers and potential therapeutic targets, circulating microRNAs and the Notch pathway represent promising avenues that could result in the early detection of the tumor and novel therapeutic approaches.

## Introduction

Malignant pleural mesothelioma (MPM) represents about 80% of mesothelioma cases. MPM is a regional and highly aggressive tumor that arises from the mesothelium of the pleural surface. Rarely, other serosal membranes of the human body are also coated with mesothelium, such as peritoneum (peritoneal mesothelioma), pericardial (pericardial mesothelioma), and tunica vaginalis (tunica vaginalis mesothelioma), are affected. Although this malignancy is rare, the incidence of MPM has increased significantly with an estimated number of about 40,000 deaths each year worldwide for asbestos-related MPM (1, 2) due to the augmented and widespread use of these carcinogenic mineral fibers (3, 4). Asbestos refers to a group of naturally occurring mineral silicate fibers with physical properties causing disease (5). The International Agency for Research on Cancer confirmed that all fibrous forms of asbestos (actinolite, amosite, anthophyllite, tremolite, crocidolite, and chrysotile) are carcinogenic to humans, causing mesothelioma. To date, asbestos includes about 400 forms of fibers that are known in nature, but among these, just the 6 forms mentioned earlier are regulated, due to their heavy commercial use. The World Health Organization estimates that 125 million people annually around the world are exposed to asbestos, both in the workplace and at home. Despite scientific evidences providing a clear and strong association between asbestos and MPM (6–9), many western countries, and newly industrializing economies, are still using asbestos (10–12). In addition, asbestos is defined differently, related to its context (commercial, mineralogical, analytical, and regulatory), and this definition has missed the cancer causing property of some minerals (5). Previous studies have reported cases of MPM in individuals exposed to erionite, regarded the most potent carcinogenic mineral fiber, but not regulated, because it is not defined as asbestos (13).

A widely accepted view assumes that the first step toward MPM is the interaction of asbestos fibers with human pleural mesothelial cells (HMC). Presumably, asbestos fibers enter the pleura and depending on the size, length of exposure, and type of deposit in different areas, cause inflammation (12), which leads to the activation of nuclear factor-kappa B (NF-κB) signaling This activation increases survival and proliferation of parietal HMC, giving rise to changes in the molecular signaling events, such as oncogenes activation, loss of tumor suppressor genes, and DNA damage, leading to an increased risk of developing MPM (14, 15). To date, the molecular mechanism(s) through which asbestos influences the selection of this HMC subpopulation(s) remains to be fully understood (16).

Several epidemiological studies demonstrated (17) an increased incidence of MPM cases among subjects, including women, with low levels or no history of occupational asbestos exposure (7). These studies indicate the existence of para-occupational exposure to asbestos, which includes exposure to asbestos workers clothes, asbestos-containing commercial products, asbestos-containing buildings, and natural asbestos in the soil, indicating that asbestos is becoming an environmental contaminant, which may act in combination with other cofactors in the MPM onset (18). Both para-occupational exposure and direct (occupational) exposure have shown to increase the risk of mesothelioma (19, 20).

In addition to asbestos exposure, other environmental interactions may increase the risk of developing MPM. Studies *in vitro* and *in vivo*, together with the detection of viral gene sequences in human specimens, have shown an association between MPM and the oncogenic simian virus 40 (SV40) (21–24), suggesting a transforming synergistic action between asbestos fibers and SV40 (25). In addition, recent immunological investigations detected a higher prevalence of SV40 antibodies in sera of MPM patients in comparison with healthy blood donors. These data strengthen the association between MPM and SV40 (26, 27). Genetic predisposition and radiation exposure seem to play a strong role as etiological factors that, alone or together with asbestos, may contribute to MPM development (17, 28, 29).

One of the peculiarities of MPM is the long-term latency period between the asbestos exposure and the tumor onset (from about 25 to 70 years), with a poor prognosis and median survival of less than 1 year from the time of diagnosis (30, 31). The majority of affected patients are 60 years old at manifestation, with peaks of the age-specific incidence at 80–84 years for men and 75–79 for women (32). In the setting of occupational asbestos exposure, the prevalence is higher among males compared with females (at male–female ratio of approximately 4:1–8:1) (7, 33).

Malignant pleural mesothelioma is heterogeneous in its histological features (34). Indeed, it can be distinguished in three main histological subtypes (35), depending both on predominant cellular component and different biological behavior. Epithelioid mesothelioma, the most common form (50–70% of cases), is characterized by polygonal, oval, or cuboidal cells similar to carcinomas; the sarcomatoid type (10–20%), with a spindle cell morphology is similar to sarcomas; while the mixed or biphasic (30%) is composed of both epithelioid and sarcomatoid forms, in different proportion, within the same tumor (36). Cytological diagnosis of MPM supported by immunohistochemistry demonstrates that the median survival varied significantly among the histological subtypes. The epithelioid subtype is less aggressive than sarcomatoid subtype. It is high sensitive and responds better to chemotherapy resulting in a longer survival than the sarcomatoid or biphasic subtypes of MPM (37, 38).

The correct identification of the MPM histological subtype facilitates the differential diagnosis, influencing subsequent prognosis and therapeutic decisions in this disease. Nevertheless, MPM is still fatal, and big efforts are being put into basic and clinical research in the attempt to find a cure for this tumor.

The aim of this review is to describe currently available therapies and to discuss novel therapeutic targets and/or early detection markers that could be developed based on the dissection of the underlying molecular mechanism involved in the onset and progression of MPM.

## Molecular Mechanisms Underlying MPM

A large number of studies carried out in the last 20 years have led to the identification of dysregulated biological processes that may play a significant role in MPM development. These studies have shown that MPM is characterized by increased cell proliferation (downregulation of tumor suppressor genes, overexpression of oncogenes), inhibition of apoptosis (39, 40), and alteration of intracellular Ca^2+^ homeostasis (41, 42). There is evidence that some of these molecular alterations, such as overexpression of adenosine A3 receptor (43), purinergic receptor P2X7 (40) and dysregulation of cellular (44) and circulating microRNAs (miRNAs) (27, 45) could be used to diagnose and interfere with MPM growth. The literature related to the most commonly found alteration in MPM is discussed below, with special emphasis on those pathways that could be exploited in the future for early diagnosis and the treatment of MPM.

### Tumor Suppressor Genes in MPM

Tumor suppressor genes play a crucial role in regulating the cell cycle. The inactivation and/or loss of their function is one of the fundamental events in the tumor development. Loss of heterozygosity, which commonly leads to unmasking a somatically mutated tumor suppressor gene through loss of the wild-type allele, seems to be a consistent feature in MPMs. Recent breakthrough studies have discovered a germline mutation/inactivation in *BAP1* (BRCA1-associated protein 1), a tumor suppressor gene located on chromosome 3p21.3 in families with a genetic predisposition to develop MPM (46, 47). *BAP1* is a deubiquitinating hydrolase that binds the RING finger domain of the BRCA1 protein, thought to be a regulator of many pathways germane to cancer (48). Previous studies reported *BAP1* involvement in various biological processes including regulation of cell cycle, response to DNA damage, and chromatin dynamics (49). BAP1 is ubiquitously expressed and interacts with tissue and cell type-specific proteins, with a role in mediating metabolic stress response (50) and in promoting survival related to its deubiquitinating activity (51). A recently published study has shown that the heterozygous germline BAP1 mutations (BAP1+/−) induce cell metabolic changes linked to the increase aerobic glycolysis, leading to reprogramming of the activities that create a favorable environment to carcinogenesis and tumor growth (52). The germline *BAP1* gene mutations lead to an abnormally short BAP1 protein that is likely broken down prematurely. These mutations have been associated with various malignancies other than malignant mesothelioma such as, uveal melanoma (47, 53) and melanocytic BAP1-associated intradermal tumors (47). Somatic truncated *BAP1* mutations and aberrant *BAP1* expression are more common in sporadic MPM, with a frequency that varies widely among different histologic tumor types (46, 54). Specifically, the pathogenesis of epithelioid subtype MPM is associated with higher survival, rather than other subtypes of malignant mesothelioma, thus providing additional clinical significance by facilitating histological classification (55–57). Besides single-point mutations in the *BAP1* gene, copy number loss, rearrangements, and multiple alterations have also been found (58, 59). Interestingly, the analysis of chromosome 3p21, using a high-density microarray-based comparative genomic hybridization (aCGH) combined with targeted next-generation DNA sequencing (NGS), detected a much higher percentage of genetic alteration in *BAP1* than reported in previous studies conducted with the NGS sequencing approach or aCGH alone, respectively. Each of these strategies resulted insufficient and less precise to identify the minute or larger chromosomal deletions, underestimating the frequency of genetic alterations in MPM (60). To date, none of mesothelioma patients with germline *BAP1* mutation was an ex-exposed asbestos worker (61), demonstrating that the development of MPM is not always directly associated with the amounts of asbestos exposure, signifying a decisive role of genetic factors among risk factors of this neoplasia.

The high incidence (around 25–60%) of the somatic *BAP1* mutations reported in MPM (62) is also associated with frequent alterations in other major tumor suppressor genes, such as *p16/Cdkn2a, p19/Arf*, and *p19/Cdkn2b* (63). Independently of *BAP1* mutations, *p16/Cdkn2A, p19/Arf*, and *p19/Cdkn2b* have been found frequently inactivated by point mutations, aberrant expression and epigenetic silencing, suggesting their role, together with asbestos exposure, in the induction of mesothelial transformation *in vitro* and *in vivo* (64). Moreover, *in vivo* studies have shown that the inactivation of both *p16* and *p19/Arf* expression accelerated the initiation of asbestos-induced MPM and decreased percent survival, as compared with the inactivation of either gene alone (65). Consistent with these data, whole-exome sequencing of asbestos-induced MPM showed the homozygous loss of *Cdkn2A* and alterations in other tumor suppressor gene (66).

Neurofibromin 2 (*NF2*) is another tumor suppressor gene frequently inactivated in MPM. A study has found that 38% of MPM samples displayed *NF2* gene mutations, and 29.4% displayed deletions, while no *NF2* mutations were found in non-small cell lung cancer patients (67). The *NF2* gene product shows a high similarity in its sequence with some members of the ERM (Ezrin, Radixin, Moesin) protein family. The NF2 protein is a scaffolding protein located at the plasma membrane, where it propagates extracellular signals through several cell surface receptors. A fraction of NF2 also interacts with other proteins that are involved in regulating ion transporters and in cytoskeletal dynamics (68).

Other studies in MPM have shown the lack of frequent mutations in two most notorious tumor suppressor genes: *p53* (64, 69) and *pRb* (70). Nevertheless, complexes between SV40 large tumor antigen protein (Tag) and both *p53* and *pRb* have been found in human mesothelioma specimens (71, 72), which results in inactivation of these important regulators of the cell proliferation and survival, thus leading to the transformation of human mesothelial cells (HM) (23, 73, 74).

### Oncogenes in MPM

Oncogenes promote transformation by driving cell proliferation and preventing apoptosis. Some of these genes are involved in the regulation of intracellular levels of calcium (Ca^2+^), an important regulator of many physiological processes, including the regulation of apoptosis of cancer cells (42, 75). The remodeling of intracellular Ca^2+^ homeostasis, as a consequence of the activity of different proteins with altered functions, is a general characteristic of cancer cells (75). It is widely accepted that both the Bcl-2 and Akt proteins are cofactors of the Ca^2+^-dependent pathways leading to apoptosis (76, 77). An antiapoptotic member of the Bcl-2 family of proteins and the oncogene *Akt* were found to be dysregulated in mesothelioma cells (78, 79), and elevated levels of Akt activity were found in 65% of human mesothelioma specimens (80, 81).

Several studies have shown that increased mesothelioma cell proliferation derives from the activity of growth factors and their specific transmembrane receptors, aberrantly expressed in human MPM (82). Epidermal growth factor receptor (*EGFR*) is an important oncogene closely involved in many cancer types, and its gene product is a transmembrane glycoprotein belonging to the tyrosine kinase receptor family. The binding between EGFR and its ligand induces cellular proliferation and cell motility and inhibits apoptosis and expression of extracellular matrix proteins (83). Previous studies have shown overexpression of *EGFR* in MPM tissues and cell lines (84, 85). A correlation between the carcinogenicity of the asbestos fibers and the induction of phosphorylation of *EGFR* was observed in rat pleural mesothelial cells (86, 87), suggesting its potential role in the pathogenesis of this cancer. Vascular endothelial growth factor (VEGF) and its receptor (VEGFR) are overexpressed in MPM human samples (88) in which they may stimulate tumor growth and promote angiogenesis and lymphangiogenesis (89, 90).

Inflammation is known to contribute to tumors by promoting cell proliferation and activating antiapoptotic pathways. The hallmarks of asbestos fibers inhalation include early and sustained inflammation linked to generation of reactive oxygen species that cause oxidative DNA damage, thus contributing to asbestos-mediated carcinogenesis (91). In addition, when asbestos fibers penetrate the pleura, HM undergo programmed cell necrosis, releasing into the extracellular space the high-mobility group box-1 (HMGB1) protein, an abundant damage-associated protein with functions linked to its cellular localization. HMGB1 mediates chronic inflammation through recruitment of macrophages, which actively secrete tumor necrosis factor-α. The pro-inflammatory and pro-survival NF-κB pathway is subsequently activated, leading to resistance to apoptosis, transformation of HM and the maintenance of the malignant phenotype (92, 93). A recent study has reported data on the high specificity of HMGB1 protein in a hyper-acetylated isoform in serum of ex-exposed mesothelioma patients, selectively discriminating against their respective healthy control. This could suggest a role for HMGB1 as a serological biomarker (94).

The major role of inflammation in MPM has been confirmed by another study showing increased concentrations of immune mediators in the sera of asbestos-exposed workers compared with controls (95). In addition, in asbestos-exposed rats, alveolar macrophages showed increased expression of transforming growth factor-β, indicating that in asbestosis these cells contribute to fibrosis as well as to an inflammatory response. Furthermore, natural killer (NK) cells demonstrated impaired cytotoxicity upon exposure to asbestos indicating that exposure to asbestos has an immune-suppressive effect, as well as a tumorigenic effect (96). Consistently, functional alteration of NK cells and cytotoxic T lymphocytes upon asbestos exposure and in MPM patients have been reported (97).

### The Oncogene Notch

The Notch signaling pathway has been found to be dysregulated in MPM human biopsies (98). In a large number of solid tumors (99–101) and leukemias (102, 103), Notch acts as an oncogene, but its role as tumor suppressor gene has been reported in other cancers, such as squamous cells carcinoma (104, 105). The Notch pathway is as a mediator of short-range cell-to-cell communication system, which involves the regulation of genes controlling developmental processes, such as proliferation, cell death, acquisition of specific cell fates, and activation of differentiation. Notch is active throughout development and during maintenance of self-renewing adult tissues, in a context-dependent manner (106). The maturation process of the Notch receptor involves a cleavage during intracellular trafficking in the Golgi complex, resulting in a single-pass transmembrane protein to shuttle to the cell membrane. The transmembrane protein is composed of a large extracellular domain linked through non covalent interactions to the transmembrane portion (107). Activation of the Notch signaling is mediated by a direct contact between the extracellular domain of Notch receptor (four members Notch1–4) and one of five canonical ligands (Delta-like 1, 3, 4 or Jagged 1, 2) on neighboring cells (108). This interaction triggers two proteolytic cleavages, initially by metalloproteases of the ADAM family, followed by a cleavage by the γ-secretase complex at the cell membrane, releasing the intracellular domain of the Notch receptor (NICD), that represents the active form of the receptor (109, 110). NICD translocates into the nucleus and interacts with the transcription factor CSL (suppressor of hairless in *Drosophila*, Lag-2 in *C. elegans*, and CBF1/RBPJ-Jκ in mammals), and, after replacing a co-repressor complex, converts it into a potent transcriptional activator of downstream target genes (111). This constitutes the “canonical” Notch pathway (106, 112). Recently, a “non-canonical” Notch pathway, which acts independently of CBF1/CSL and plays important roles in normal and transformed cells, has been identified (113, 114).

There is a functional diversity among the Notch receptors, in particular among their intracellular active forms that are capable of inducing specific genes (115–117). There is evidence that, in breast carcinoma, Notch2 has opposite effects on cell survival, compared with Notch1 and Notch4 (118). Furthermore, it has been observed that the transcriptional activity of Notch1 and Notch3 is reduced by co-expression with the intracellular domain of Notch2 (119). A detailed description of the biochemical processes regulated by Notch and the implications of dysregulation of this pathway in the development of cancer have been widely discussed and reviewed elsewhere (106, 120–123).

In cell lines established from human MPM biopsies, elevated Notch1 and reduced Notch2 expression have been observed (98) with their normal counterparts. Genetic and chemical modulation of the Notch pathway indicated that MPM cells are dependent on Notch signaling. Specifically, in MPM cells, Notch 1 inhibits PTEN (phosphatase and tensin homolog) and activates the PI3K/Akt/mTOR signaling pathway indicating that this receptor is an MPM oncogene and its activation is strictly necessary for growth and survival of MPM cells (98). On the contrary, in MPM cells, Notch2 is a positive transcriptional regulation of PTEN and therefore an inhibitor of the PI3K/Akt/mTOR signaling pathway and re-expression of Notch-2 was toxic to MPM cells (98). Previous studies conducted by the same group have shown that SV40 activates Notch1 leading to immortalization and transformation of primary HMC (124–126). These data indicate that Notch1 can mediate the process of transformation of mesothelial cells, downstream of mutagenic events caused by the exposure of carcinogenic factors, such as asbestos and viral infection (125, 127–129). The effect of SV40 on Notch1 in mesothelial cells is similar to what reported in uterine cervical cancer, in which the infection of human papilloma virus has been linked to the activation of Notch1 (101, 130, 131).

## Current Therapeutic Approaches to MPM

### Surgical Treatment

Surgery, also used in combination with chemo- and/or radiotherapy, attempts to eradicate the malignant tissue and is an essential option to help the patient relieve symptoms by reducing pain and by controlling pleural effusions (132). Nevertheless, surgical resection of the tumor is controversial and limited to MPM patients with early stage disease and good cardiopulmonary functions (133, 134). The intent and the role of surgical procedure influence the survival rate of MPM patients. In the analysis of the International Association for the study of Lung Cancer Mesothelioma Database (3, 101), MPM patients undergoing curative-intent surgery had a median survival of 18 months (stage I, 21 months; stage II, 19 months; stage III, 16 months; and stage IV, 12 months) vs 12 months with the palliative intent (135). A large study (14,288 patients) has shown that surgery alone, compared with no treatment, is associated with a significant improvement in survival [adjusted hazard ratio (adj HR) 0.64 (0.61–0.67)], but not radiation [adj HR 1.15 (1.08–1.23)]. The similar survival obtained with surgery alone has been observed after surgery and radiation combined [adj HR 0.69 (0.64–0.76)] (136). There are two surgical procedures commonly used in MPM: (1) pleurectomy/decortication (P/D) that involves the radical removal of all visible disease of the pleura, both the inner and outer lung lining. If the mesothelioma only affects one lung, a pneumonectomy may be performed to remove the entire organ; (2) extra-pleural pneumonectomy (EPP), a type of more radical surgical option which aims to eradicate all macroscopic tumors *via* the removal of the areas surrounding it, including other mesothelial tissue (137). The optimal procedure for resection (EPP or P/D) of MPM is controversial and depends on clinical factors and on individual surgical preference and expertise. Flores et al. have shown that operative mortality following EPP is higher compared with P/D (7 vs 4%, respectively); however, P/D has better survival, compared with EPP (16 vs 12 months, respectively). All things considered, the authors of the study highlight similarities between the two approaches and conclude that there is no evidence to support the use of EPP vs P/D (138). A multicenter randomized clinical trial [Mesothelioma and Radical Surgery (MARS)] compared the clinical outcomes between MPM patients assigned to EPP within trimodal therapy (chemotherapy, EPP, and postoperative hemithorax irradiation) and patients with chemotherapy, but no EPP. The median survival for the EPP group was 14.4 months, while for the no EPP group was 19.5 months (139). The higher mortality related to EPP (3–15%) compared with extended P/D (1–5%) and, the observation that both techniques can achieve prolonged median survival has been reported by other groups (140). When balancing these considerations has been emphasized that surgery should be aimed to obtain macroscopic compete resection while limiting surgery-associated mortality and given the high rates of local failure/recurrence after surgery, incorporation of intracavitary therapeutics into the multimodality treatments (MMT) protocol would be desirable (140).

### Radiotherapy

Radiation therapy is relatively common for MPM. Several studies have shown that radiotherapy is unable to cure MPM (141), but administrated either pre- or postoperatively, in combination with other treatments or alone, is useful to control pain, limit tumor spreading and, only in combination with other approaches, improved the 2-year rate of overall survival from 20 to 34% (142). It has proven to be extremely difficult to identify the effective radiation dose and the site of the radiation, due to the unique way that MPM spreads along the pleura, surrounding the lungs, adjacent to the heart, the spinal, and other vital organs. Once the cancerous cells spread, they can form small tumor called nodules. This process, known as seeding, may occur in 20–50% of MPM patients. Prophylactic radiation has been used to prevent spreading and procedure-tract metastases, but this approach remains controversial and without a standardized clinical practice, due to mixed results obtained (143). Differences in surgical procedures, closely related to the ability to administer radiation, could explain the mixed results (144). In the neoadjuvant setting, the development of new intensity-modulated radiation therapy (IMRT), followed by early EPP, allowed the optimization of the administration of high-dose radiotherapy to the hemithorax, providing in selected MPM patients an improved median overall survival up to 39.4 months (145, 146). In contrast with these results, multicenter clinical trials observed the not promising outcomes of IMRT after adjuvant chemotherapy and EPP, not supporting the routine use of hemithoracic therapy for MPM in trimodality approach, due to the high toxic effects (147). The concerns related to the radiation treatment in the trimodality approach were the high rate of patients excluded, due to disease progression, surgical mortality, hemithoracic radiation morbidity, and not satisfactory risk/benefit ratio (148). With the lack of randomized trials, Wald and Sugarbaker recommends the application of radiation following EPP surgical procedure to selected patients with good postoperative recovery, excluding those patients who had local chest wall invasion (140).

### Chemotherapy

Despite the toxic effects of chemical drugs, systemic chemotherapy for MPM remains the only and primary treatment modality and reasonable option that has been shown to increase median survival from 9 to 12 months in most advanced stage MPM patients, who are not candidates for aggressive surgery (149). Almost every chemotherapy regimen has been tested in mesothelioma (150, 151). Although these treatments are no curative, they can alleviate symptoms, improve quality of life and prolong survival, depending upon the tumor stage, histological differentiation, and the patient’s overall health when treatment begins (132). Platinum containing regimens have a greater activity than non-platinum containing combinations (152). Vogelzang et al. were the first to demonstrate that pemetrexed/cisplatin combination chemotherapy is more effective in MPM than cisplatin monotherapy (153, 154). A few other combinations were evaluated in randomized trials, but they did not demonstrate an important improvement of overall survival (155). Of note early clinical trials of MPM patients included heterogeneous groups of patients with divergent risk factors and were therefore often not powerful enough to assess therapeutic efficacy of a particular treatment (156). New generation of antifolates (pemetrexed and raltitrexed) and novel platinum derivatives (157) have shown low efficacy and limited outcomes, with a 3 months survival benefit in their combination over cisplatin alone: the median survival ranged from 9 to 12 months, as shown by the EMPHACIS trial in patients with advanced disease (153).

There are still many unanswered questions regarding chemotherapy and MPM. However, chemotherapy remains a central pillar of systemic therapy for MPM and the goal today is still to develop novel targeted chemotherapy agents, to be used either alone or in combination to increase efficacy and to minimize and/or avoid side effects (157). A number of novel therapeutic agents are under investigation with the aim to provide further treatment options for MPM in the future (158).

### Multimodality Therapy

According to the 2007 UK Department of Health’s Mesothelioma Service Framework and the British Thoracic Society’s Statement on Mesothelioma (159), progress has been made in the management of MPM patients by involving experienced multidisciplinary team recommended by other guidelines for mesothelioma patients (160, 161). For more effective results, treatment options include the combination of two or more different methods of treatment, such as surgery, radiation therapy, and chemotherapy. However, timing, type of agent, and modality still debated. Selected patients with operable disease and a good performance status should be considered candidates for the multimodality therapy. For instance, surgery is recommended for patients with clinical stage I disease where the tumor is localized and non-metastatic to lymph nodes or other organs or tissues and has potential for surgical tolerance. Patients who are not operable because impaired cardiopulmonary function can be treated with chemotherapy. Patients with stage II where the tumor is larger and has invaded nearby organs, such as the lung or diaphragm, lymph nodes, may also be involved and stage III where mesothelioma has invaded a region or area, such as the chest wall, esophagus, or lymph nodes should be offered trimodal therapy with surgery, chemotherapy, and radiotherapy. Chemotherapy alone is recommended for patients who are not medically fit for surgery have stage IV disease and/or show sarcomatoid histology (162). A recent study has confirmed that the combination of surgical treatment, such as EPP and chemotherapy with radiotherapy led to a median survival that ranged from 18 to 24 months (163). In the MARS study, radical EPP within trimodal therapy showed no benefits on the quality life overall survival, compared with chemotherapy (139). Wald and Sugarbaker discussed that without clinical studies comparing both chemotherapy or chemoradiotherapy to surgery-based MMT protocols and different surgery-based MMT approaches, there remains considerable uncertainty about the right therapeutic protocol and the right type of surgery for the individual patient (140). In agreement with their observation, data from the Cochrane Lung Cancer group’s Specialized Register, Cochrane Central Register of Controlled Trials, Medline, Embase, and the strength of the evidence collected by Abdel-Rahman et al. revealed that there is still a lack of available evidence to support the use of radical multimodality therapy in routine clinical practice (164).

### Immunotherapy and Targeted Therapy

As for other cancer types, immunotherapy is opening new options for the treatment of MPM. Clinical trials with dendritic cells and live-attenuated *Listeria* vaccination have produced encouraging results and are being considered for multicenter phase II trials (165). Intrapleural injection of oncolytic viruses (herpesvirus, poxvirus, adenovirus, and several attenuated RNA viruses) has also been considered as a possible treatment for unresectable MPM, due to the sensibility of MPM cells to their action, by direct killing or by immune-mediated mechanisms (166). In the light of the high levels of treatment-related adverse events or limited benefits of immunotherapeutic approaches in many clinical trials, the application of oncolytic virotherapy in MPM treatment is still being investigated (167).

Clinical trials are also being conducted to test effectiveness of immune checkpoint inhibitors in MPM patients. In a large clinical study, treatment with tremelimumab, a monoclonal antibody against cytotoxic-T-lymphocyte-associated antigen 4, expressed on the surface of activated T lymphocytes and interfering with their ability to kill cancer cells did not significantly prolong overall survival, compared with placebo (median survival of 7.3 months) in patients with previously treated MPM (median survival of 7.7 months) (168). Safety of pembrolizumab, an antibody against PD-L1 (programmed cell death ligand 1), and an inhibitor of immune response expressed on cancer cells have also been tested in MPM patients. It appears to be well tolerated, and it might confer antitumor activity in patients with PD-L1-positive MPM. Response, durability, and efficacy in this patient population warrant further investigation (169). Nevertheless, one of the biggest trials for immune checkpoint blockade has reported death of 81% of MPM patients died, without significant difference in overall survival between therapeutic treatments against placebo (168).

A promising type of immunotherapy based on adoptive cell transfer employs chimeric antigen receptor (CAR) T cells, in which T cells are generated to recognize specific antigen receptors (TSA or TAAs) on cancer cells. Numerous trials are currently being explored for MPM. Specifically, phase I clinical trials for genetically modified T cells to recognize specific antigens on MPM cells, such as mesothelin and fibroblast activation protein (FPA), are being conducted in MPM patients (170).

In preclinical setting, the dysregulation in MPM of ErbB, a protein structurally similar and a ligand to EGFR, is being exploited for immunotherapy (171). To this aim, patient T-cells were engineered by retroviral transduction to express a panErbB-targeted CAR and co-expressed with a chimeric cytokine receptor, to induce interleukin-4 mediated CAR T-cell proliferation. These cells were activated upon contact with a panel of four mesothelioma cell lines, leading to cytotoxicity and cytokine release in all cases (171). Preclinical studies are also providing proof of concept that combination treatment of chemotherapy/radiotherapy and immunotherapy with immune checkpoint inhibitors could lead to better outcomes for MPM. Specifically, it has been demonstrated *in vivo* that short course of high-dose non-ablative radiation could promote an antitumor immune response (165).

In the context of targeted therapy, phase I and phase II trials, which tested inhibitors of receptors with tyrosine kinase activity (RTK) in MPM patients with dysregulated of EGFR and VEGFR pathways have given poor results (172). The best results to date are with combination of bevacizumab and chemotherapy leading to 2.6 months increase of survival when compared with patients who did not receive bevacizumab (173). Inactivation of plasminogen activators inhibitor PAI-1, implicated in tumor progression by increasing angiogenesis, could constitute a strategy for inhibiting angiogenesis and growth of MPM. In a preclinical setting, tumor mass and the degree of angiogenesis in intrapleural tumors were reduced when PAI-1 inhibitor was administered to mice in which MPM cells expressing high levels of VEGF (VEGF-A) which were intrapleurally transplanted (174).

A detailed list of the ongoing clinical trials based on immunotherapy has been recently published (167). Based on the recent results of the studies conducted so far Bakker and colleagues conclude that this therapeutic approach for MPM has been disappointing probably due to the chronic inflammatory state and hypoxia that define this tumor. To further complicate the immune-therapeutic approach to MPM, it has been recently shown that MPM is a pool of independent clones that would need to be simultaneously targeted for an effective immunotherapy (175).

## New Therapeutic Approaches and Novel Molecular Targets

Despite progresses, survival time and response rate to cytotoxic agents used for MPM treatment are still not satisfactory (176), plus a high degree of variability in treatment outcome in cancer patients undergoing chemotherapy has been observed (177). Furthermore, the cancer is still diagnosed at an advanced stage. Therefore, there is a strong need for early and accurate diagnosis markers and new therapeutic approaches in MPM.

### Circulating Biomarkers of MPM

Analysis of liquid samples, such as serum and pleural effusion, due to their ease of collection represents a promising approach for the characterization of markers related to cancer progression (178). Recently, proteins (179–183), metabolites (184), and miRNAs (45) have been identified which are differentially expressed in the serum of MPM patients and could be used as biomarkers of the onset and progression of MPM. However, these studies still require a definitive validation in larger populations.

Soluble mesothelin is a cell surface glycoprotein highly expressed in several human cancers, including mesothelioma (185). Several studies have shown a sensitivity of 84% for advanced status of MPM, a specificity of 95%, and a correlation with histological subtype of the tumor (186–188). The MPM patients with epithelioid subtype had higher levels of mesothelin than those with sarcomatoid subtype (189). Another highly promising biomarker is the circulating glycoprotein fibulin-3 (190). A study population conducted by Pass et al. showed elevated fibulin-3 levels both in plasma (specificity of 94% and sensitivity of 100%) and pleural effusion (specificity of 93% and sensitivity of 84%) of MPM patients, distinguishing healthy persons with exposure to asbestos from patients with MPM (180). The prognostic potential of fibulin-3 is superior compared with mesothelin, which instead results more useful as diagnostic biomarker of MPM (181). Pass and colleagues have also shown that the osteopontin levels, an extracellular cell adhesion protein, were significantly higher in serum of MPM patients than healthy asbestos-exposed individuals (179). However, it has been observed that osteopontin is unable to distinguish between MPM, pleural metastatic carcinoma or benign pleural lesion, associated with asbestos exposure, due to very high number of false-positive (191).

A clinical study has shown that total or hyper-acetylated isoform of HMGB1 is a sensitive and specific biomarker that allows to differentiate early the serum samples of MPM patient asbestos-exposed from healthy unexposed ones and other pleural diseases patients (94). In subjects from a hyperendemic area for MPM, the C–C chemokine Regulated on Activation, Normal T-cell-Expressed and Secreted (RANTES) was found associated with the exposure to asbestos fibers. RANTES showed an increased gradient of concentration from healthy subjects to asbestos-exposed workers and MPM patients. Independently of SV40 infection, increased concentrations of other immune mediators were observed in the sera of the asbestos-exposed workers compared with controls (95). Analyses carried out on serum samples from MPM patients have detected the presence of antibodies against SV40 viral capsid protein (27, 192–194). Since SV40 could synergize with asbestos in causing MPM (21, 125), these antibodies in the serum could help to predict the risk of developing MPM in a population of asbestos-exposed worker.

The discovery of miRNAs, which are small sequences of RNA involved in regulation of gene expression, has changed the approach to diagnosis and therapy of many diseases, including cancer (44). miRNAs regulate a plethora of cellular activities, such as proliferation, apoptosis, metabolism, and angiogenesis. They are characterized by high stability, under different conditions and typology of sample treatment, processing, and isolation (195–197). Furthermore, these circulating miRNAs, moving though the circulatory system naked or inside microparticles, such as exosomes, microvescicles, and apoptotic bodies, represent an innovative form of distant intracellular communication (45, 198, 199). The miRNAs expression profile has been found to be abnormal in several human cancers, thus pointing at their role in cancer progression, as oncomiRNAs and tumor suppressor miRNAs (200–202). Based on their characteristics of measurable indicators of physiological and pathological conditions, miRNAs could be used for prognosis, diagnosis, and treatment outcome assessments of cancers, including the MPM (8, 45).

A specific circulating miRNAs signature discriminating MPM patients from ex-exposed asbestos and healthy subjects have been identified (27, 45, 203). It has been proposed that the detection of circulating miRNAs, i.e., miR-197-3p, miR-1281, and miR-32-3p, in sera of MPM affected patients and workers ex-exposed to asbestos fibers could be used as a novel, non-invasive, predictive biomarkers for this cancer (45). This signature could also help to design targeted therapies for MPM (8, 204), exploiting the use of antagomir (oligonucleotide sequences) or anti-miRNAs (mimetic miRNA) (44), to silence the overexpressed oncomiRs or substitute the lost miRNA in cancer, respectively (205, 206).

### Targeting the Notch Pathway in MPM

A large body of evidence shows that inhibition of Notch signaling causes a reduction of tumor cell proliferation *in vitro* and arrests tumor growth *in vivo* (99, 207), thus the targeting of Notch offers an attractive potential therapeutic strategy in oncology. Notch inhibition is able to shrink the tumor not only by increasing the apoptotic rate in the bulk of tumor but also by inhibiting the growth of cancer stem cells, one main culprit for tumor recurrence (208), and by interfering with angiogenesis (209). Small molecules inhibiting γ-secretase, the enzyme required for Notch activation are being investigated in clinical trials (for a list of trials the reader is referred to http://clinicaltrials.gov). The molecules seem to work best in combination treatment (210). Other agents able to reduce angiogenesis by inhibiting Notch in are also being developed, i.e., antibodies against Dll4 (211, 212).

As previously discussed, Notch1 is overexpressed in MPM and it is therefore possible to envision the targeting of this receptor, in combination with other agents already used to prevent MPM progression by interfering with angiogenesis (172) and cancer stem cells survival (213). The efficacy of Notch inhibitors could be evaluated also in experimental model of MPM in which less known pathways crucial for mesothelioma stem cells, such as Wnt (91, 214) and Hyppos, downstream of *NF2* (215), are being investigated. The rationale for this co-treatment is based on evidence of the cross talks between the Notch, Hyppos, and Wnt pathways (210).

Targeting Notch in tumors has been challenging due to the fact that γ-secretase have many side effects (101). In this context, MPM may be a good model to test the efficacy of novel therapies that target Notch1 because of its location in a closed space (the pleural cavity) that could limit the toxicity due to γ-secretase inhibition. It should be noted that targeting Notch in tumor has been challenging also due to the complexity of Notch signaling. First, not all Notch receptors are sensitive to inhibition of γ-secretase (115) and second, whereas canonical Notch signaling is very well documented in cancer, the non-canonical activation of Notch signaling, which plays a role in cancer, in still not well understood (216, 217). Consequently, it is not always possible to assess Notch inhibition by a certain agent. The clear dissection of the canonical and non-canonical Notch signaling is necessary to fully understand this complex pathway to develop a targeted therapy that includes Notch.

### Precision Oncology and Future Directions in the Treatments of MPM Patients

**Figure 1:**
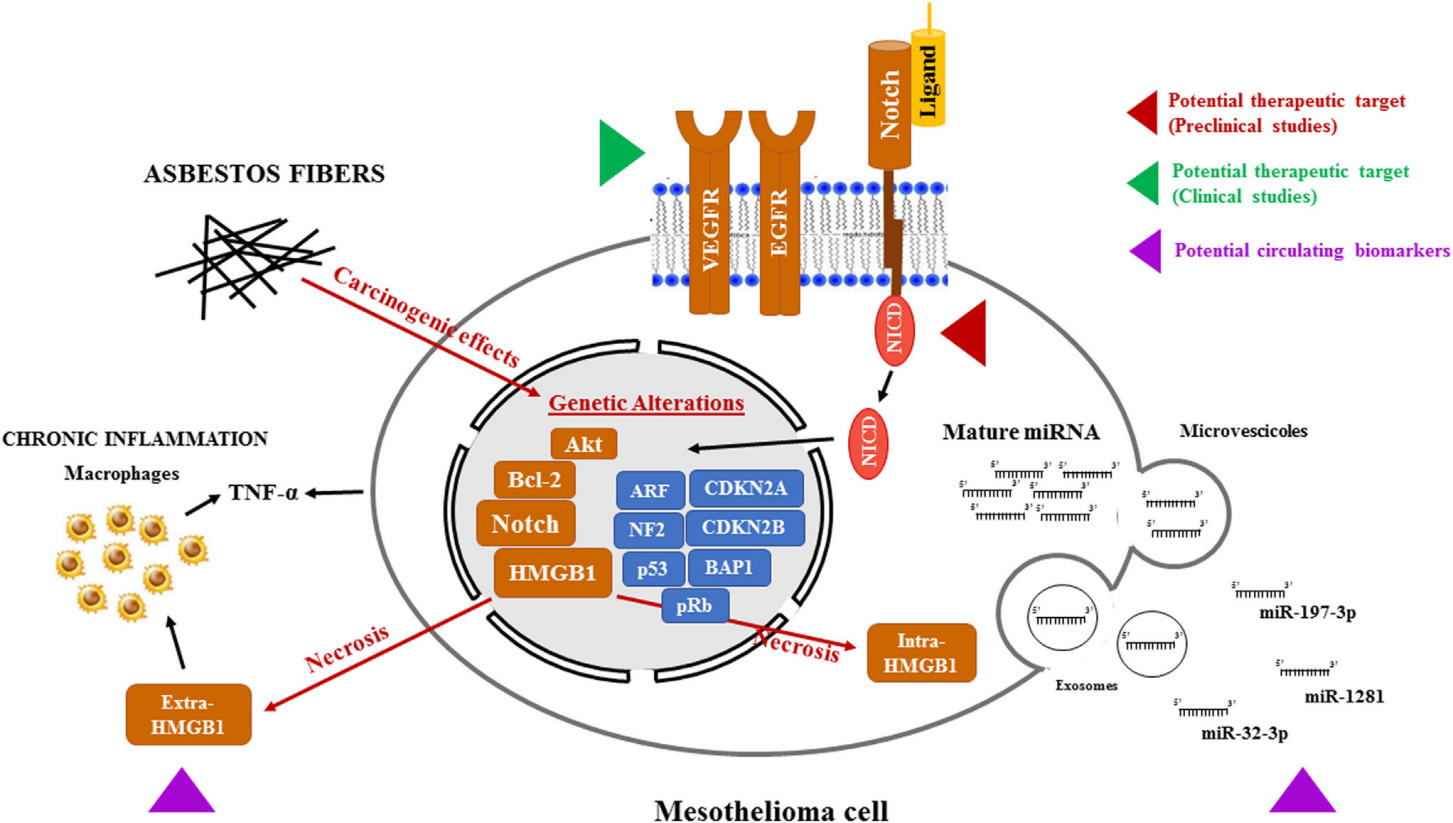
Potential therapeutic targets and biomarkers in the mesothelioma. Asbestos fibers, alone or with other cofactors, such as viral infection or genetic predisposition, may cause mutagenic changes resulting in the alterations of oncogenes and tumor suppressor genes leading to the transformation of normal mesothelial cells. In addition, following necrosis caused by asbestos exposure, HMGB1 primarily located in the nucleus, translocates to the cytosol and extracellular space, triggering the inflammatory response and TNF-α secretion, both by mesothelial cells and macrophages, further contributing to mesothelial cells transformation. All these events may contribute to the activation of the Notch signaling. Targeting Notch, as it is already been pursued with VEGFR and EGFR, could help to stop the progression of mesothelioma. Mesothelioma is also accompanied by changes in microRNA (miRNA) expression in cancer cells and, consequently, in biological fluids. In particular, miRNAs (miR-197-3p, miR-1281, and miR-32-3p) could become a tool for early diagnosis of mesothelioma. In this figure, the red and green arrows represent clinical and preclinical studies, respectively, aimed to sensitize mesothelioma cells to cytotoxic treatments by targeting newly discovered pathways altered in mesothelioma. The purple arrow indicates the novel potential circulating biomarkers under study for a no invasive MPM screening. Abbreviations: VEGFR, vascular endothelial growth factor receptor; EGFR, epidermal growth factor receptor; HMGB1, high-mobility group box-1; TNF-α, tumor necrosis factor-α; MPM, malignant pleural mesothelioma.

Studies conducted in the last decades have identified several pathways that could be targeted to give new hopes to MPM patients (Figure 1). Due to the rapidly evolving field of precision medicine, identification of novel biomarkers is promising as it may provide the best therapeutic options for each patient, considered the genetic background and the specific characteristics of the tumor. This hope is supported by a recent study on polymorphisms in gemcitabine, pemetrexed, and cisplatin metabolism, transport and other drug target genes and DNA repair pathways, which has led to a clinical-pharmacogenetic model that could predict the best chemotherapeutic treatment for a specific MPM patient (177). Furthermore, oncogene-targeted depth sequencing on a tumor sample and paired peripheral blood DNA from a patient with malignant mesothelioma of the peritoneum revealed a mutation leading to 13-amino acids neo-peptide of the truncated BAP1 protein, which is predicted to be present on this examined patient’s HLA-B protein. This tumor-specific neoantigen is an example of potential molecular biomarker for personalized diagnosis of mesothelioma (177, 218). Comprehensive genomic analysis, followed by integrated analyses of 216 MPM biopsies, has identified recurrent mutations, gene fusions and splicing alterations leading to inactivation of *NF2, BAP1*, and *SETD2* and alterations in Hippo, mTOR, histone methylation, RNA helicase, and p53 signaling pathways (219). Furthermore, in a proof-of-concept study including five MPM patients, it was shown that the composition of pleural effusion is dynamic, influenced by treatment and that the immune cell composition of the pleural effusion does not automatically reflect the properties of tumor tissue. These findings could have major consequences when applying precision immunotherapy based on pleural effusion findings in patients (199). In conclusion, with the aim to achieve early detection of MPM and increased survival of these patients, with minimal side effects: (1) the comprehensive genomic profiling of MPM; (2) the targeting of pathways already known to be dysregulated in MPMs, such as the Notch pathway; (3) the characterization of dysregulated circulating miRNAs, and (4) the assessment of risks, such as exposure to asbestos and the presence of germline BAP1 mutations, should all be taken under consideration.

## Author Contributions

PR and MR participated in the designing of the manuscript and draft it. IB, AC, and RF participated in drafting the manuscript and critically revised it. FM and MT designed and coordinated the manuscript. All the authors read and approved the final manuscript.

## Conflict of Interest Statement

The authors declare that the research was conducted in the absence of any commercial or financial relationships that could be construed as a potential conflict of interest. The reviewer MC and the handling Editor declared their shared affiliation.

## References

[1] Driscoll T Nelson DI Steenland K Leigh J Concha-Barrientos M Fingerhut M The global burden of disease due to occupational carcinogens. Am J Ind Med (2005) 48(6):419–31. 10.1002/ajim.20209 16299703

[2] Grondin SC Sugarbaker DJ Malignant mesothelioma of the pleural space. Oncology (Williston Park) (1999) 13(7):919–26; discussion 26, 31–2. 10442339

[3] Mao W Zhang X Guo Z Gao Z Pass HI Yang H Association of asbestos exposure with malignant mesothelioma incidence in Eastern China. JAMA Oncol (2017) 3(4):562–4. 10.1001/jamaoncol.2016.5487 27918607 PMC5569880

[4] Carbone M Kanodia S Chao A Miller A Wali A Weissman D Consensus report of the 2015 Weinman international conference on mesothelioma. J Thorac Oncol (2016) 11(8):1246–62. 10.1016/j.jtho.2016.04.028 27453164 PMC5551435

[5] Baumann F Ambrosi JP Carbone M Asbestos is not just asbestos: an unrecognised health hazard. Lancet Oncol (2013) 14(7):576–8. 10.1016/S1470-2045(13)70257-2 23725699

[6] Qi F Okimoto G Jube S Napolitano A Pass HI Laczko R Continuous exposure to chrysotile asbestos can cause transformation of human mesothelial cells via HMGB1 and TNF-alpha signaling. Am J Pathol (2013) 183(5):1654–66. 10.1016/j.ajpath.2013.07.029 24160326 PMC3814524

[7] Baumann F Buck BJ Metcalf RV McLaurin BT Merkler DJ Carbone M. The presence of asbestos in the natural environment is likely related to mesothelioma in young individuals and women from Southern Nevada. J Thorac Oncol (2015) 10(5):731–7. 10.1097/JTO.0000000000000506 25668121 PMC4406807

[8] Chen Z Gaudino G Pass HI Carbone M Yang H. Diagnostic and prognostic biomarkers for malignant mesothelioma: an update. Transl Lung Cancer Res (2017) 6(3):259–69. 10.21037/tlcr.2017.05.06 28713671 PMC5504120

[9] Carbone M Bedrossian CW. The pathogenesis of mesothelioma. Semin Diagn Pathol (2006) 23(1):56–60. 10.1053/j.semdp.2006.08.002 17044196

[10] Baumann F Flores E Napolitano A Kanodia S Taioli E Pass H Mesothelioma patients with germline BAP1 mutations have 7-fold improved long-term survival. Carcinogenesis (2015) 36(1):76–81. 10.1093/carcin/bgu227 25380601 PMC4291047

[11] Jaklitsch MT Grondin SC Sugarbaker DJ. Treatment of malignant mesothelioma. World J Surg (2001) 25(2):210–7. 10.1007/s002680020021 11338024

[12] Kadariya Y Menges CW Talarchek J Cai KQ Klein-Szanto AJ Pietrofesa RA Inflammation-related IL1beta/IL1R signaling promotes the development of asbestos-induced malignant mesothelioma. Cancer Prev Res (Phila) (2016) 9(5):406–14. 10.1158/1940-6207.CAPR-15-0347 26935421 PMC4854753

[13] Carbone M Baris YI Bertino P Brass B Comertpay S Dogan AU Erionite exposure in North Dakota and Turkish villages with mesothelioma. Proc Natl Acad Sci U S A (2011) 108(33):13618–23. 10.1073/pnas.1105887108 21788493 PMC3158231

[14] Yang H Bocchetta M Kroczynska B Elmishad AG Chen Y Liu Z TNF-alpha inhibits asbestos-induced cytotoxicity via a NF-kappaB-dependent pathway, a possible mechanism for asbestos-induced oncogenesis. Proc Natl Acad Sci U S A (2006) 103(27):10397–402. 10.1073/pnas.0604008103 16798876 PMC1502469

[15] Sartore-Bianchi A Gasparri F Galvani A Nici L Darnowski JW Barbone D Bortezomib inhibits nuclear factor-kappaB dependent survival and has potent in vivo activity in mesothelioma. Clin Cancer Res (2007) 13(19):5942–51. 10.1158/1078-0432.CCR-07-0536 17908991

[16] Singh A Pruett N Hoang CD. In vitro experimental models of mesothelioma revisited. Transl Lung Cancer Res (2017) 6(3):248–58. 10.21037/tlcr.2017.04.12 28713670 PMC5504106

[17] Carbone M Yang H. Mesothelioma: recent highlights. Ann Transl Med (2017) 5(11):238. 10.21037/atm.2017.04.29 28706906 PMC5497108

[18] Bolognesi C Martini F Tognon M Filiberti R Neri M Perrone E A molecular epidemiology case control study on pleural malignant mesothelioma. Cancer Epidemiol Biomarkers Prev (2005) 14(7):1741–6. 10.1158/1055-9965.EPI-04-0903 16030111

[19] Roggli VL Sharma A Butnor KJ Sporn T Vollmer RT. Malignant mesothelioma and occupational exposure to asbestos: a clinicopathological correlation of 1445 cases. Ultrastruct Pathol (2002) 26(2):55–65. 10.1080/01913120252959227 12036093

[20] Noonan CW. Environmental asbestos exposure and risk of mesothelioma. Ann Transl Med (2017) 5(11):234. 10.21037/atm.2017.03.74 28706902 PMC5497111

[21] Cristaudo A Foddis R Vivaldi A Buselli R Gattini V Guglielmi G SV40 enhances the risk of malignant mesothelioma among people exposed to asbestos: a molecular epidemiologic case-control study. Cancer Res (2005) 65(8):3049–52. 10.1158/0008-5472.CAN-04-2219 15833832

[22] Carbone M Pannuti A Zhang L Testa JR Bocchetta M. A novel mechanism of late gene silencing drives SV40 transformation of human mesothelial cells. Cancer Res (2008) 68(22):9488–96. 10.1158/0008-5472.CAN-08-2332 19010924 PMC2666620

[23] Barbanti-Brodano G Sabbioni S Martini F Negrini M Corallini A Tognon M. Simian virus 40 infection in humans and association with human diseases: results and hypotheses. Virology (2004) 318(1):1–9. 10.1016/j.virol.2003.09.004 15015494

[24] Gazdar AF Carbone M. Molecular pathogenesis of malignant mesothelioma and its relationship to simian virus 40. Clin Lung Cancer (2003) 5(3):177–81. 10.3816/CLC.2003.n.031 14667274

[25] Kroczynska B Cutrone R Bocchetta M Yang H Elmishad AG Vacek P Crocidolite asbestos and SV40 are cocarcinogens in human mesothelial cells and in causing mesothelioma in hamsters. Proc Natl Acad Sci U S A (2006) 103(38):14128–33. 10.1073/pnas.0604544103 16966607 PMC1599923

[26] Carbone M Rizzo P Pass H. Simian virus 40: the link with human malignant mesothelioma is well established. Anticancer Res (2000) 20(2A):875–7. 10810369

[27] Mazzoni E Corallini A Cristaudo A Taronna A Tassi G Manfrini M High prevalence of serum antibodies reacting with simian virus 40 capsid protein mimotopes in patients affected by malignant pleural mesothelioma. Proc Natl Acad Sci U S A (2012) 109(44):18066–71. 10.1073/pnas.1213238109 23071320 PMC3497789

[28] Ohar JA Cheung M Talarchek J Howard SE Howard TD Hesdorffer M Germline BAP1 mutational landscape of asbestos-exposed malignant mesothelioma patients with family history of cancer. Cancer Res (2016) 76(2):206–15. 10.1158/0008-5472.CAN-15-0295 26719535 PMC4715907

[29] Goodman JE Nascarella MA Valberg PA. Ionizing radiation: a risk factor for mesothelioma. Cancer Causes Control (2009) 20(8):1237–54. 10.1007/s10552-009-9357-4 19444627

[30] Carbone M Ly BH Dodson RF Pagano I Morris PT Dogan UA Malignant mesothelioma: facts, myths, and hypotheses. J Cell Physiol (2012) 227(1):44–58. 10.1002/jcp.22724 21412769 PMC3143206

[31] Carbone M Kratzke RA Testa JR. The pathogenesis of mesothelioma. Semin Oncol (2002) 29(1):2–17. 10.1053/sonc.2002.30227 11836664

[32] Robinson BM Malignant pleural mesothelioma: an epidemiological perspective. Ann Cardiothorac Surg (2012) 1(4):491–6. 10.3978/j.issn.2225-319X.2012.11.04 23977542 PMC3741803

[33] Yang H Testa JR Carbone M. Mesothelioma epidemiology, carcinogenesis, and pathogenesis. Curr Treat Options Oncol (2008) 9(2–3):147–57. 10.1007/s11864-008-0067-z 18709470 PMC2717086

[34] Husain AN Colby TV Ordonez NG Krausz T Borczuk A Cagle PT Guidelines for pathologic diagnosis of malignant mesothelioma: a consensus statement from the International Mesothelioma Interest Group. Arch Pathol Lab Med (2009) 133(8):1317–31. 10.1043/1543-2165-133.8.1317 19653732

[35] Husain AN Colby TV Ordonez NG Allen TC Attanoos RL Beasley MB Guidelines for pathologic diagnosis of malignant mesothelioma: 2017 update of the consensus statement from the International Mesothelioma Interest Group. Arch Pathol Lab Med (2017) 142(1):10–142. 10.5858/arpa.2017-0124-RA 28686500

[36] Geltner C Errhalt P Baumgartner B Ambrosch G Machan B Eckmayr J Management of malignant pleural mesothelioma – part 1: epidemiology, diagnosis, and staging: consensus of the Austrian Mesothelioma Interest Group (AMIG). Wien Klin Wochenschr (2016) 128(17–18):611–7. 10.1007/s00508-016-1080-z 27619223 PMC5033998

[37] Brims FJ Meniawy TM Duffus I de Fonseka D Segal A Creaney J A novel clinical prediction model for prognosis in malignant pleural mesothelioma using decision tree analysis. J Thorac Oncol (2016) 11(4):573–82. 10.1016/j.jtho.2015.12.108 26776867

[38] Bille A Krug LM Woo KM Rusch VW Zauderer MG. Contemporary analysis of prognostic factors in patients with unresectable malignant pleural mesothelioma. J Thorac Oncol (2016) 11(2):249–55. 10.1016/j.jtho.2015.10.003 26845118 PMC4917284

[39] Missiroli S Bonora M Patergnani S Poletti F Perrone M Gafa R PML at mitochondria-associated membranes is critical for the repression of autophagy and cancer development. Cell Rep (2016) 16(9):2415–27. 10.1016/j.celrep.2016.07.082 27545895 PMC5011426

[40] Amoroso F Salaro E Falzoni S Chiozzi P Giuliani AL Cavallesco G P2X7 targeting inhibits growth of human mesothelioma. Oncotarget (2016) 7(31):49664–76. 10.18632/oncotarget.10430 27391069 PMC5226537

[41] Bononi A Giorgi C Patergnani S Larson D Verbruggen K Tanji M BAP1 regulates IP3R3-mediated Ca^2+^ flux to mitochondria suppressing cell transformation. Nature (2017) 546(7659):549–53. 10.1038/nature22798 28614305 PMC5581194

[42] Patergnani S Giorgi C Maniero S Missiroli S Maniscalco P Bononi I The endoplasmic reticulum mitochondrial calcium cross talk is downregulated in malignant pleural mesothelioma cells and plays a critical role in apoptosis inhibition. Oncotarget (2015) 6(27):23427–44. 10.18632/oncotarget.4370 26156019 PMC4695128

[43] Varani K Maniero S Vincenzi F Targa M Stefanelli A Maniscalco P A(3) receptors are overexpressed in pleura from patients with mesothelioma and reduce cell growth via Akt/nuclear factor-kappaB pathway. Am J Respir Crit Care Med (2011) 183(4):522–30. 10.1164/rccm.201006-0980OC 20870754

[44] Balatti V Maniero S Ferracin M Veronese A Negrini M Ferrocci G MicroRNAs dysregulation in human malignant pleural mesothelioma. J Thorac Oncol (2011) 6(5):844–51. 10.1097/JTO.0b013e31820db125 21358347

[45] Bononi I Comar M Puozzo A Stendardo M Boschetto P Orecchia S Circulating microRNAs found dysregulated in ex-exposed asbestos workers and pleural mesothelioma patients as potential new biomarkers. Oncotarget (2016) 7(50):82700–11. 10.18632/oncotarget.12408 27716620 PMC5347725

[46] Testa JR Cheung M Pei J Below JE Tan Y Sementino E Germline BAP1 mutations predispose to malignant mesothelioma. Nat Genet (2011) 43(10):1022–5. 10.1038/ng.912 21874000 PMC3184199

[47] Carbone M Ferris LK Baumann F Napolitano A Lum CA Flores EG BAP1 cancer syndrome: malignant mesothelioma, uveal and cutaneous melanoma, and MBAITs. J Transl Med (2012) 10:179. 10.1186/1479-5876-10-179 22935333 PMC3493315

[48] Carbone M Yang H Pass HI Krausz T Testa JR Gaudino G. BAP1 and cancer. Nat Rev Cancer (2013) 13(3):153–9. 10.1038/nrc3459 23550303 PMC3792854

[49] Yu H Pak H Hammond-Martel I Ghram M Rodrigue A Daou S Tumor suppressor and deubiquitinase BAP1 promotes DNA double-strand break repair. Proc Natl Acad Sci U S A (2014) 111(1):285–90. 10.1073/pnas.1309085110 24347639 PMC3890818

[50] Baughman JM Rose CM Kolumam G Webster JD Wilkerson EM Merrill AE NeuCode proteomics reveals Bap1 regulation of metabolism. Cell Rep (2016) 16(2):583–95. 10.1016/j.celrep.2016.05.096 27373151 PMC5546211

[51] Dai F Lee H Zhang Y Zhuang L Yao H Xi Y BAP1 inhibits the ER stress gene regulatory network and modulates metabolic stress response. Proc Natl Acad Sci U S A (2017) 114(12):3192–7. 10.1073/pnas.1619588114 28275095 PMC5373337

[52] Bononi A Yang H Giorgi C Patergnani S Pellegrini L Su M Germline BAP1 mutations induce a Warburg effect. Cell Death Differ (2017) 24(10):1694–704. 10.1038/cdd.2017.95 28665402 PMC5596430

[53] Harbour JW Onken MD Roberson ED Duan S Cao L Worley LA Frequent mutation of BAP1 in metastasizing uveal melanomas. Science (2010) 330(6009):1410–3. 10.1126/science.1194472 21051595 PMC3087380

[54] Nasu M Emi M Pastorino S Tanji M Powers A Luk H High incidence of somatic BAP1 alterations in sporadic malignant mesothelioma. J Thorac Oncol (2015) 10(4):565–76. 10.1097/JTO.0000000000000471 25658628 PMC4408084

[55] Yoshikawa Y Sato A Tsujimura T Emi M Morinaga T Fukuoka K Frequent inactivation of the BAP1 gene in epithelioid-type malignant mesothelioma. Cancer Sci (2012) 103(5):868–74. 10.1111/j.1349-7006.2012.02223.x 22321046 PMC7659203

[56] Bononi A Napolitano A Pass HI Yang H Carbone M. Latest developments in our understanding of the pathogenesis of mesothelioma and the design of targeted therapies. Expert Rev Respir Med (2015) 9(5):633–54. 10.1586/17476348.2015.1081066 26308799 PMC4887271

[57] McGregor SM Dunning R Hyjek E Vigneswaran W Husain AN Krausz T. BAP1 facilitates diagnostic objectivity, classification, and prognostication in malignant pleural mesothelioma. Hum Pathol (2015) 46(11):1670–8. 10.1016/j.humpath.2015.06.024 26376834

[58] Shinozaki-Ushiku A Ushiku T Morita S Anraku M Nakajima J Fukayama M. Diagnostic utility of BAP1 and EZH2 expression in malignant mesothelioma. Histopathology (2017) 70(5):722–33. 10.1111/his.13123 27859460

[59] Righi L Duregon E Vatrano S Izzo S Giorcelli J Rondon-Lagos M BRCA1-associated protein 1 (BAP1) immunohistochemical expression as a diagnostic tool in malignant pleural mesothelioma classification: a large retrospective study. J Thorac Oncol (2016) 11(11):2006–17. 10.1016/j.jtho.2016.06.020 27422796

[60] Yoshikawa Y Emi M Hashimoto-Tamaoki T Ohmuraya M Sato A Tsujimura T High-density array-CGH with targeted NGS unmask multiple noncontiguous minute deletions on chromosome 3p21 in mesothelioma. Proc Natl Acad Sci U S A (2016) 113(47):13432–7. 10.1073/pnas.1612074113 27834213 PMC5127333

[61] Carbone M Flores EG Emi M Johnson TA Tsunoda T Behner D Combined genetic and genealogic studies uncover a large BAP1 cancer syndrome kindred tracing back nine generations to a common ancestor from the 1700s. PLoS Genet (2015) 11(12):e1005633. 10.1371/journal.pgen.1005633 26683624 PMC4686043

[62] Xu J Kadariya Y Cheung M Pei J Talarchek J Sementino E Germline mutation of Bap1 accelerates development of asbestos-induced malignant mesothelioma. Cancer Res (2014) 74(16):4388–97. 10.1158/0008-5472.CAN-14-1328 24928783 PMC4165574

[63] Cheng JQ Jhanwar SC Klein WM Bell DW Lee WC Altomare DA p16 alterations and deletion mapping of 9p21–p22 in malignant mesothelioma. Cancer Res (1994) 54(21):5547–51. 7923195

[64] Lecomte C Andujar P Renier A Kheuang L Abramowski V Mellottee L Similar tumor suppressor gene alteration profiles in asbestos-induced murine and human mesothelioma. Cell Cycle (2005) 4(12):1862–9. 10.4161/cc.4.12.2300 16319530

[65] Altomare DA Menges CW Xu J Pei J Zhang L Tadevosyan A Losses of both products of the Cdkn2a/Arf locus contribute to asbestos-induced mesothelioma development and cooperate to accelerate tumorigenesis. PLoS One (2011) 6(4):e18828. 10.1371/journal.pone.0018828 21526190 PMC3079727

[66] Sneddon S Patch AM Dick IM Kazakoff S Pearson JV Waddell N Whole exome sequencing of an asbestos-induced wild-type murine model of malignant mesothelioma. BMC Cancer (2017) 17(1):396. 10.1186/s12885-017-3382-6 28577549 PMC5455120

[67] Andujar P Pairon JC Renier A Descatha A Hysi I Abd-Alsamad I Differential mutation profiles and similar intronic TP53 polymorphisms in asbestos-related lung cancer and pleural mesothelioma. Mutagenesis (2013) 28(3):323–31. 10.1093/mutage/get008 23435014

[68] Beltrami S Kim R Gordon J. Neurofibromatosis type 2 protein, NF2: an uncoventional cell cycle regulator. Anticancer Res (2013) 33(1):1–11. 23267122 PMC3725758

[69] Kumar K Rahman Q Schipper H Matschegewski C Schiffmann D Papp T. Mutational analysis of 9 different tumour-associated genes in human malignant mesothelioma cell lines. Oncol Rep (2005) 14(3):743–50. 10.3892/or.14.3.743 16077986

[70] Lee AY Raz DJ He B Jablons DM. Update on the molecular biology of malignant mesothelioma. Cancer (2007) 109(8):1454–61. 10.1002/cncr.22552 17348013

[71] Carbone M Rizzo P Grimley PM Procopio A Mew DJ Shridhar V Simian virus-40 large-T antigen binds p53 in human mesotheliomas. Nat Med (1997) 3(8):908–12. 10.1038/nm0897-908 9256284

[72] De Luca A Baldi A Esposito V Howard CM Bagella L Rizzo P The retinoblastoma gene family pRb/p105, p107, pRb2/p130 and simian virus-40 large T-antigen in human mesotheliomas. Nat Med (1997) 3(8):913–6. 10.1038/nm0897-913 9256285

[73] Tognon M Martini F Corallini A Barbanti-Brodano G SV40 and human cancers. Int J Cancer (2004) 110(5):778–9; author reply 80. 10.1002/ijc.20150 15146570

[74] Rizzo P Bocchetta M Powers A Foddis R Stekala E Pass HI SV40 and the pathogenesis of mesothelioma. Semin Cancer Biol (2001) 11(1):63–71. 10.1006/scbi.2000.0347 11243900

[75] Marchi S Pinton P. Alterations of calcium homeostasis in cancer cells. Curr Opin Pharmacol (2016) 29:1–6. 10.1016/j.coph.2016.03.002 27043073

[76] Marchi S Rimessi A Giorgi C Baldini C Ferroni L Rizzuto R Akt kinase reducing endoplasmic reticulum Ca^2+^ release protects cells from Ca^2+^-dependent apoptotic stimuli. Biochem Biophys Res Commun (2008) 375(4):501–5. 10.1016/j.bbrc.2008.07.153 18723000 PMC2576286

[77] Pinton P Ferrari D Rapizzi E Di Virgilio F Pozzan T Rizzuto R. The Ca^2+^ concentration of the endoplasmic reticulum is a key determinant of ceramide-induced apoptosis: significance for the molecular mechanism of Bcl-2 action. EMBO J (2001) 20(11):2690–701. 10.1093/emboj/20.11.2690 11387204 PMC125256

[78] Cioce M Canino C Goparaju C Yang H Carbone M Pass HI. Autocrine CSF-1R signaling drives mesothelioma chemoresistance via AKT activation. Cell Death Dis (2014) 5:e1167. 10.1038/cddis.2014.136 24722292 PMC5424113

[79] Braun F de Carne Trecesson S Bertin-Ciftci J Juin P. Protect and serve: Bcl-2 proteins as guardians and rulers of cancer cell survival. Cell Cycle (2013) 12(18):2937–47. 10.4161/cc.25972 23974114 PMC3875667

[80] Altomare DA You H Xiao GH Ramos-Nino ME Skele KL De Rienzo A Human and mouse mesotheliomas exhibit elevated AKT/PKB activity, which can be targeted pharmacologically to inhibit tumor cell growth. Oncogene (2005) 24(40):6080–9. 10.1038/sj.onc.1208744 15897870

[81] Pinton G Manente AG Angeli G Mutti L Moro L. Perifosine as a potential novel anti-cancer agent inhibits EGFR/MET-AKT axis in malignant pleural mesothelioma. PLoS One (2012) 7(5):e36856. 10.1371/journal.pone.0036856 22590625 PMC3349630

[82] Zhou S Liu L Li H Eilers G Kuang Y Shi S Multipoint targeting of the PI3K/mTOR pathway in mesothelioma. Br J Cancer (2014) 110(10):2479–88. 10.1038/bjc.2014.220 24762959 PMC4021537

[83] Ciardiello F Tortora G. A novel approach in the treatment of cancer: targeting the epidermal growth factor receptor. Clin Cancer Res (2001) 7(10):2958–70. 11595683

[84] Janne PA Taffaro ML Salgia R Johnson BE. Inhibition of epidermal growth factor receptor signaling in malignant pleural mesothelioma. Cancer Res (2002) 62(18):5242–7. 12234991

[85] Huang L Cai M Zhang X Wang F Chen L Xu M Combinational therapy of crizotinib and afatinib for malignant pleural mesothelioma. Am J Cancer Res (2017) 7(2):203–17. 28337371 PMC5336496

[86] Faux SP Houghton CE. Cell signaling in mesothelial cells by asbestos: evidence for the involvement of oxidative stress in the regulation of the epidermal growth factor receptor. Inhal Toxicol (2000) 12(Suppl 3):327–36. 10.1080/08958378.2000.11463242 26368632

[87] Zanella CL Posada J Tritton TR Mossman BT. Asbestos causes stimulation of the extracellular signal-regulated kinase 1 mitogen-activated protein kinase cascade after phosphorylation of the epidermal growth factor receptor. Cancer Res (1996) 56(23):5334–8. 8968079

[88] Aoe K Hiraki A Tanaka T Gemba K Taguchi K Murakami T Expression of vascular endothelial growth factor in malignant mesothelioma. Anticancer Res (2006) 26(6C):4833–6. 17214348

[89] Strizzi L Catalano A Vianale G Orecchia S Casalini A Tassi G Vascular endothelial growth factor is an autocrine growth factor in human malignant mesothelioma. J Pathol (2001) 193(4):468–75. 10.1002/path.824 11276005

[90] Ohta Y Shridhar V Bright RK Kalemkerian GP Du W Carbone M VEGF and VEGF type C play an important role in angiogenesis and lymphangiogenesis in human malignant mesothelioma tumours. Br J Cancer (1999) 81(1):54–61. 10.1038/sj.bjc.6690650 10487612 PMC2374345

[91] Carbone M Yang H. Molecular pathways: targeting mechanisms of asbestos and erionite carcinogenesis in mesothelioma. Clin Cancer Res (2012) 18(3):598–604. 10.1158/1078-0432.CCR-11-2259 22065079 PMC3291331

[92] Pellegrini L Xue J Larson D Pastorino S Jube S Forest KH HMGB1 targeting by ethyl pyruvate suppresses malignant phenotype of human mesothelioma. Oncotarget (2017) 8(14):22649–61. 10.18632/oncotarget.15152 28186988 PMC5410252

[93] Jube S Rivera ZS Bianchi ME Powers A Wang E Pagano I Cancer cell secretion of the DAMP protein HMGB1 supports progression in malignant mesothelioma. Cancer Res (2012) 72(13):3290–301. 10.1158/0008-5472.CAN-11-3481 22552293 PMC3389268

[94] Napolitano A Antoine DJ Pellegrini L Baumann F Pagano I Pastorino S HMGB1 and its hyperacetylated isoform are sensitive and specific serum biomarkers to detect asbestos exposure and to identify mesothelioma patients. Clin Cancer Res (2016) 22(12):3087–96. 10.1158/1078-0432.CCR-15-1130 26733616 PMC4867109

[95] Comar M Zanotta N Bonotti A Tognon M Negro C Cristaudo A Increased levels of C-C chemokine RANTES in asbestos exposed workers and in malignant mesothelioma patients from an hyperendemic area. PLoS One (2014) 9(8):e104848. 10.1371/journal.pone.0104848 25162674 PMC4146505

[96] Nishimura Y Maeda M Kumagai-Takei N Lee S Matsuzaki H Wada Y Altered functions of alveolar macrophages and NK cells involved in asbestos-related diseases. Environ Health Prev Med (2013) 18(3):198–204. 10.1007/s12199-013-0333-y 23463177 PMC3650181

[97] Nishimura Y Kumagai-Takei N Matsuzaki H Lee S Maeda M Kishimoto T Functional alteration of natural killer cells and cytotoxic T lymphocytes upon asbestos exposure and in malignant mesothelioma patients. Biomed Res Int (2015) 2015:238431. 10.1155/2015/238431 26161391 PMC4486484

[98] Graziani I Eliasz S De Marco MA Chen Y Pass HI De May RM Opposite effects of Notch-1 and Notch-2 on mesothelioma cell survival under hypoxia are exerted through the Akt pathway. Cancer Res (2008) 68(23):9678–85. 10.1158/0008-5472.CAN-08-0969 19047145

[99] Rizzo P Osipo C Foreman K Golde T Osborne B Miele L. Rational targeting of Notch signaling in cancer. Oncogene (2008) 27(38):5124–31. 10.1038/onc.2008.226 18758481

[100] Crabtree JS Singleton CS Miele L. Notch signaling in neuroendocrine tumors. Front Oncol (2016) 6:94. 10.3389/fonc.2016.00094 27148486 PMC4830836

[101] Espinoza I Miele L. Notch inhibitors for cancer treatment. Pharmacol Ther (2013) 139(2):95–110. 10.1016/j.pharmthera.2013.02.003 23458608 PMC3732476

[102] Gu Y Masiero M Banham AH. Notch signaling: its roles and therapeutic potential in hematological malignancies. Oncotarget (2016) 7(20):29804–23. 10.18632/oncotarget.7772 26934331 PMC5045435

[103] Kushwah R Guezguez B Lee JB Hopkins CI Bhatia M. Pleiotropic roles of Notch signaling in normal, malignant, and developmental hematopoiesis in the human. EMBO Rep (2014) 15(11):1128–38. 10.15252/embr.201438842 25252682 PMC4253487

[104] Nicolas M Wolfer A Raj K Kummer JA Mill P van Noort M Notch1 functions as a tumor suppressor in mouse skin. Nat Genet (2003) 33(3):416–21. 10.1038/ng1099 12590261

[105] Nowell CS Radtke F. Notch as a tumour suppressor. Nat Rev Cancer (2017) 17(3):145–59. 10.1038/nrc.2016.145 28154375

[106] Kopan R Ilagan MX. The canonical Notch signaling pathway: unfolding the activation mechanism. Cell (2009) 137(2):216–33. 10.1016/j.cell.2009.03.045 19379690 PMC2827930

[107] Gordon WR Arnett KL Blacklow SC The molecular logic of Notch signaling – a structural and biochemical perspective. J Cell Sci (2008) 121(Pt 19):3109–19. 10.1242/jcs.035683 18799787 PMC2696053

[108] Siebel C Lendahl U. Notch signaling in development, tissue homeostasis, and disease. Physiol Rev (2017) 97(4):1235–94. 10.1152/physrev.00005.2017 28794168

[109] Artavanis-Tsakonas S Rand MD Lake RJ. Notch signaling: cell fate control and signal integration in development. Science (1999) 284(5415):770–6. 10.1126/science.284.5415.770 10221902

[110] Ray WJ Yao M Mumm J Schroeter EH Saftig P Wolfe M Cell surface presenilin-1 participates in the gamma-secretase-like proteolysis of Notch. J Biol Chem (1999) 274(51):36801–7. 10.1074/jbc.274.51.36801 10593990

[111] Nam Y Weng AP Aster JC Blacklow SC. Structural requirements for assembly of the CSL.intracellular Notch1.Mastermind-like 1 transcriptional activation complex. J Biol Chem (2003) 278(23):21232–9. 10.1074/jbc.M301567200 12644465

[112] D’Souza B Meloty-Kapella L Weinmaster G. Canonical and non-canonical Notch ligands. Curr Top Dev Biol (2010) 92:73–129. 10.1016/S0070-2153(10)92003-6 20816393 PMC4286395

[113] Ayaz F Osborne BA. Non-canonical notch signaling in cancer and immunity. Front Oncol (2014) 4:345. 10.3389/fonc.2014.00345 25538890 PMC4255497

[114] Traustadottir GA Jensen CH Garcia Ramirez JJ Beck HC Sheikh SP Andersen DC. The non-canonical NOTCH1 ligand Delta-like 1 homolog (DLK1) self interacts in mammals. Int J Biol Macromol (2017) 97:460–7. 10.1016/j.ijbiomac.2017.01.067 28099888

[115] Fortini F Vieceli Dalla Sega F Caliceti C Aquila G Pannella M Pannuti A Estrogen receptor beta-dependent Notch1 activation protects vascular endothelium against tumor necrosis factor alpha (TNFalpha)-induced apoptosis. J Biol Chem (2017) 292(44):18178–91. 10.1074/jbc.M117.790121 28893903 PMC5672041

[116] Previs RA Coleman RL Harris AL Sood AK Molecular pathways: translational and therapeutic implications of the Notch signaling pathway in cancer. Clin Cancer Res (2015) 21(5):955–61. 10.1158/1078-0432.CCR-14-0809 25388163 PMC4333206

[117] Vieceli Dalla Sega F Aquila G Fortini F Vaccarezza M Secchiero P Rizzo P Context-dependent function of ROS in the vascular endothelium: the role of the Notch pathway and shear stress. Biofactors (2017) 43(4):475–85. 10.1002/biof.1359 28419584

[118] O’Neill CF Urs S Cinelli C Lincoln A Nadeau RJ Leon R Notch2 signaling induces apoptosis and inhibits human MDA-MB-231 xenograft growth. Am J Pathol (2007) 171(3):1023–36. 10.2353/ajpath.2007.061029 17675579 PMC1959488

[119] Shimizu K Chiba S Saito T Kumano K Hamada Y Hirai H. Functional diversity among Notch1, Notch2, and Notch3 receptors. Biochem Biophys Res Commun (2002) 291(4):775–9. 10.1006/bbrc.2002.6528 11866432

[120] Yao Y Ni Y Zhang J Wang H Shao S. The role of Notch signaling in gastric carcinoma: molecular pathogenesis and novel therapeutic targets. Oncotarget (2017) 8(32):53839–53. 10.18632/oncotarget.17809 28881855 PMC5581154

[121] O’Brien R Marignol L. The Notch-1 receptor in prostate tumorigenesis. Cancer Treat Rev (2017) 56:36–46. 10.1016/j.ctrv.2017.04.003 28457880

[122] Aster JC Pear WS Blacklow SC. The varied roles of Notch in cancer. Annu Rev Pathol (2017) 12:245–75. 10.1146/annurev-pathol-052016-100127 27959635 PMC5933931

[123] Fortini ME Bilder D. Endocytic regulation of Notch signaling. Curr Opin Genet Dev (2009) 19(4):323–8. 10.1016/j.gde.2009.04.005 19447603 PMC2731830

[124] Carbone M Bocchetta M SV40 and Notch-I: multi-functionality meets pleiotropy. Prog Mol Subcell Biol (2004) 36:289–305. 10.1007/978-3-540-74264-7_14 15171617

[125] Bocchetta M Miele L Pass HI Carbone M. Notch-1 induction, a novel activity of SV40 required for growth of SV40-transformed human mesothelial cells. Oncogene (2003) 22(1):81–9. 10.1038/sj.onc.1206097 12527910

[126] Bocchetta M Di Resta I Powers A Fresco R Tosolini A Testa JR Human mesothelial cells are unusually susceptible to simian virus 40-mediated transformation and asbestos cocarcinogenicity. Proc Natl Acad Sci U S A (2000) 97(18):10214–9. 10.1073/pnas.170207097 10954737 PMC27818

[127] Carbone M Burck C Rdzanek M Rudzinski J Cutrone R Bocchetta M. Different susceptibility of human mesothelial cells to polyomavirus infection and malignant transformation. Cancer Res (2003) 63(19):6125–9. 14559789

[128] Kroczynska B Carbone M. Cross reactivity between many anti-human antibodies for their hamster homologs provide the tools to study the signal transduction pathway activated by asbestos and SV40 in the malignant mesothelioma model. Mol Carcinog (2006) 45(7):537–42. 10.1002/mc.20200 16649249

[129] Thompson JK MacPherson MB Beuschel SL Shukla A. Asbestos-induced mesothelial to fibroblastic transition is modulated by the inflammasome. Am J Pathol (2017) 187(3):665–78. 10.1016/j.ajpath.2016.11.008 28056339 PMC5389358

[130] Clementz AG Rizzo P Martini F Tognon M Roles of dysregulated Notch pathway and small DNA tumor viruses in cancer initiation and progression. J Cancer Metastasis Treat (2016) 2:11–23. 10.4103/2394-4722.171982

[131] Lathion S Schaper J Beard P Raj K. Notch1 can contribute to viral-induced transformation of primary human keratinocytes. Cancer Res (2003) 63(24):8687–94. 14695182

[132] Bibby AC Tsim S Kanellakis N Ball H Talbot DC Blyth KG Malignant pleural mesothelioma: an update on investigation, diagnosis and treatment. Eur Respir Rev (2016) 25(142):472–86. 10.1183/16000617.0063-2016 27903668 PMC9487555

[133] Cao C Tian D Park J Allan J Pataky KA Yan TD. A systematic review and meta-analysis of surgical treatments for malignant pleural mesothelioma. Lung Cancer (2014) 83(2):240–5. 10.1016/j.lungcan.2013.11.026 24360321

[134] Wald O Sugarbaker DJ Perspective on malignant pleural mesothelioma diagnosis and treatment. Ann Transl Med (2016) 4(6):120 10.21037/atm.2016.03.17 27127773 PMC4828731

[135] Rusch VW Giroux D Kennedy C Ruffini E Cangir AK Rice D Initial analysis of the international association for the study of lung cancer mesothelioma database. J Thorac Oncol (2012) 7(11):1631–9. 10.1097/JTO.0b013e31826915f1 23070243

[136] Taioli E Wolf AS Camacho-Rivera M Kaufman A Lee DS Nicastri D Determinants of survival in malignant pleural mesothelioma: a surveillance, epidemiology, and end results (SEER) study of 14,228 patients. PLoS One (2015) 10(12):e0145039. 10.1371/journal.pone.0145039 26660351 PMC4682765

[137] Butchart EG Ashcroft T Barnsley WC Holden MP. Pleuropneumonectomy in the management of diffuse malignant mesothelioma of the pleura. Experience with 29 patients. Thorax (1976) 31(1):15–24. 10.1136/thx.31.1.15 1257933 PMC470356

[138] Flores RM Pass HI Seshan VE Dycoco J Zakowski M Carbone M Extrapleural pneumonectomy versus pleurectomy/decortication in the surgical management of malignant pleural mesothelioma: results in 663 patients. J Thorac Cardiovasc Surg (2008) 135(3):620–6, 6.e1–3. 10.1016/j.jtcvs.2007.10.054 18329481

[139] Treasure T Lang-Lazdunski L Waller D Bliss JM Tan C Entwisle J Extra-pleural pneumonectomy versus no extra-pleural pneumonectomy for patients with malignant pleural mesothelioma: clinical outcomes of the mesothelioma and radical surgery (MARS) randomised feasibility study. Lancet Oncol (2011) 12(8):763–72. 10.1016/S1470-2045(11)70149-8 21723781 PMC3148430

[140] Wald O Sugarbaker DJ. New concepts in the treatment of malignant pleural mesothelioma. Annu Rev Med (2018) 69:365–77. 10.1146/annurev-med-041316-085813 29029582

[141] Ung YC Yu E Falkson C Haynes AE Stys-Norman D Evans WK The role of radiation therapy in malignant pleural mesothelioma: a systematic review. Radiother Oncol (2006) 80(1):13–8. 10.1016/j.radonc.2006.06.002 16820238

[142] Rosenzweig KE. Malignant pleural mesothelioma: adjuvant therapy with radiation therapy. Ann Transl Med (2017) 5(11):242. 10.21037/atm.2017.06.25 28706910 PMC5497112

[143] Clive AO Taylor H Dobson L Wilson P de Winton E Panakis N Prophylactic radiotherapy for the prevention of procedure-tract metastases after surgical and large-bore pleural procedures in malignant pleural mesothelioma (SMART): a multicentre, open-label, phase 3, randomised controlled trial. Lancet Oncol (2016) 17(8):1094–104. 10.1016/S1470-2045(16)30095-X 27345639 PMC4961873

[144] Hiddinga BI Rolfo C van Meerbeeck JP. Mesothelioma treatment: are we on target? A review. J Adv Res (2015) 6(3):319–30. 10.1016/j.jare.2014.11.012 26257929 PMC4522581

[145] Opitz I. Management of malignant pleural mesothelioma-the European experience. J Thorac Dis (2014) 6(Suppl 2):S238–52. 10.3978/j.issn.2072-1439.2014.05.03 24868442 PMC4032963

[146] Perrot M Wu L Wu M Cho BCJ. Radiotherapy for the treatment of malignant pleural mesothelioma. Lancet Oncol (2017) 18(9):e532–42. 10.1016/S1470-2045(17)30459-X 28884702

[147] Stahel RA Riesterer O Xyrafas A Opitz I Beyeler M Ochsenbein A Neoadjuvant chemotherapy and extrapleural pneumonectomy of malignant pleural mesothelioma with or without hemithoracic radiotherapy (SAKK 17/04): a randomised, international, multicentre phase 2 trial. Lancet Oncol (2015) 16(16):1651–8. 10.1016/S1470-2045(15)00208-9 26538423

[148] Hasegawa S Okada M Tanaka F Yamanaka T Soejima T Kamikonya N Trimodality strategy for treating malignant pleural mesothelioma: results of a feasibility study of induction pemetrexed plus cisplatin followed by extrapleural pneumonectomy and postoperative hemithoracic radiation (Japan Mesothelioma Interest Group 0601 Trial). Int J Clin Oncol (2016) 21(3):523–30. 10.1007/s10147-015-0925-1 26577445 PMC4901093

[149] Nowak AK Chemotherapy for malignant pleural mesothelioma: a review of current management and a look to the future. Ann Cardiothorac Surg (2012) 1(4):508–15. 10.3978/j.issn.2225-319X.2012.10.05 23977545 PMC3741799

[150] Janne PA. Chemotherapy for malignant pleural mesothelioma. Clin Lung Cancer (2003) 5(2):98–106. 10.3816/CLC.2003.n.023 14596692

[151] Tomek S Manegold C. Chemotherapy for malignant pleural mesothelioma: past results and recent developments. Lung Cancer (2004) 45(Suppl 1):S103–19. 10.1016/j.lungcan.2004.04.020 15261443

[152] Kelly K Azzoli CG Zatloukal P Albert I Jiang PY Bodkin D Randomized phase 2b study of pralatrexate versus erlotinib in patients with stage IIIB/IV non-small-cell lung cancer (NSCLC) after failure of prior platinum-based therapy. J Thorac Oncol (2012) 7(6):1041–8. 10.1097/JTO.0b013e31824cc66c 22534814

[153] Vogelzang NJ Rusthoven JJ Symanowski J Denham C Kaukel E Ruffie P Phase III study of pemetrexed in combination with cisplatin versus cisplatin alone in patients with malignant pleural mesothelioma. J Clin Oncol (2003) 21(14):2636–44. 10.1200/JCO.2003.11.136 12860938

[154] van Meerbeeck JP Gaafar R Manegold C Van Klaveren RJ Van Marck EA Vincent M Randomized phase III study of cisplatin with or without raltitrexed in patients with malignant pleural mesothelioma: an intergroup study of the European Organisation for Research and Treatment of Cancer Lung Cancer Group and the National Cancer Institute of Canada. J Clin Oncol (2005) 23(28):6881–9. 10.1200/JCO.20005.14.589 16192580

[155] Blomberg C Nilsson J Holgersson G Edlund P Bergqvist M Adwall L Randomized trials of systemic medically-treated malignant mesothelioma: a systematic review. Anticancer Res (2015) 35(5):2493–501. 25964522

[156] Curran D Sahmoud T Therasse P van Meerbeeck J Postmus PE Giaccone G. Prognostic factors in patients with pleural mesothelioma: the European Organization for Research and Treatment of Cancer experience. J Clin Oncol (1998) 16(1):145–52. 10.1200/JCO.1998.16.1.145 9440736

[157] Garland LL. Chemotherapy for malignant pleural mesothelioma. Curr Treat Options Oncol (2011) 12(2):181–8. 10.1007/s11864-011-0152-6 21468683

[158] Tsao AS Moon J Wistuba II Vogelzang NJ Kalemkerian GP Redman MW Phase I trial of cediranib in combination with cisplatin and pemetrexed in chemonaive patients with unresectable malignant pleural mesothelioma (SWOG S0905). J Thorac Oncol (2017) 12(8):1299–308. 10.1016/j.jtho.2017.05.021 28599887 PMC5690479

[159] British Thoracic Society Standards of Care Committee. BTS statement on malignant mesothelioma in the UK, 2007. Thorax (2007) 62(Suppl 2):ii1–19. 10.1136/thx.2007.087619 17965072 PMC2094726

[160] Baas P Fennell D Kerr KM Van Schil PE Haas RL Peters S Malignant pleural mesothelioma: ESMO clinical practice guidelines for diagnosis, treatment and follow-up. Ann Oncol (2015) 26(Suppl 5):v31–9. 10.1093/annonc/mdv199 26223247

[161] Ettinger DS Wood DE Akerley W Bazhenova LA Borghaei H Camidge DR NCCN guidelines insights: malignant pleural mesothelioma, version 3.2016. J Natl Compr Canc Netw (2016) 14(7):825–36. 10.6004/jnccn.2016.0087 27407123 PMC10187059

[162] Mott FE Mesothelioma: a review. Ochsner J (2012) 12(1):70–9. 22438785 PMC3307510

[163] Kishimoto T Fujimoto N Nishi H [Clinical pathological diagnosis, and treatment for pleural mesothelioma]. Gan To Kagaku Ryoho (2016) 43(5):513–7. 27210080

[164] Abdel-Rahman O Elsayed Z Mohamed H Eltobgy M. Radical multimodality therapy for malignant pleural mesothelioma. Cochrane Database Syst Rev (2018) 1:CD012605. 10.1002/14651858.CD012605.pub2 29309720 PMC6491325

[165] Wu L de Perrot M. Radio-immunotherapy and chemo-immunotherapy as a novel treatment paradigm in malignant pleural mesothelioma. Transl Lung Cancer Res (2017) 6(3):325–34. 10.21037/tlcr.2017.06.03 28713677 PMC5504113

[166] Boisgerault N Achard C Delaunay T Cellerin L Tangy F Gregoire M Oncolytic virotherapy for human malignant mesothelioma: recent advances. Oncolytic Virother (2015) 4:133–40. 10.2147/OV.S66091 27512676 PMC4918388

[167] Bakker E Guazzelli A Ashtiani F Demonacosb C Krstic-Demonacos M Mutti L Immunotherapy advances for mesothelioma treatment. Expert Rev Anticanc (2017) 17(9):799–814. 10.1080/14737140.2017.1358091 28724330

[168] Maio M Scherpereel A Calabro L Aerts J Perez SC Bearz A Tremelimumab as second-line or third-line treatment in relapsed malignant mesothelioma (DETERMINE): a multicentre, international, randomised, double-blind, placebo-controlled phase 2b trial. Lancet Oncol (2017) 18(9):1261–73. 10.1016/S1470-2045(17)30446-1 28729154

[169] Alley EW Lopez J Santoro A Morosky A Saraf S Piperdi B Clinical safety and activity of pembrolizumab in patients with malignant pleural mesothelioma (KEYNOTE-028): preliminary results from a non-randomised, open-label, phase 1b trial. Lancet Oncol (2017) 18(5):623–30. 10.1016/S1470-2045(17)30169-9 28291584

[170] Zeltsman M Dozier J McGee E Ngai D Adusumilli PS. CAR T-cell therapy for lung cancer and malignant pleural mesothelioma. Transl Res (2017) 187:1–10. 10.1016/j.trsl.2017.04.004 28502785 PMC5581988

[171] Klampatsa A Achkova DY Davies DM Parente-Pereira AC Woodman N Rosekilly J Intracavitary ‘T4 immunotherapy’ of malignant mesothelioma using pan-ErbB re-targeted CAR T-cells. Cancer Lett (2017) 393:52–9. 10.1016/j.canlet.2017.02.015 28223167

[172] Guazzelli A Bakker E Tian K Demonacos C Krstic-Demonacos M Mutti L. Promising investigational drug candidates in phase I and phase II clinical trials for mesothelioma. Expert Opin Investig Drugs (2017) 26(8):933–44. 10.1080/13543784.2017.1351545 28679291

[173] Zalcman G Mazieres J Margery J Greillier L Audigier-Valette C Moro-Sibilot D Bevacizumab for newly diagnosed pleural mesothelioma in the Mesothelioma Avastin Cisplatin Pemetrexed Study (MAPS): a randomised, controlled, open-label, phase 3 trial. Lancet (2016) 387(10026):1405–14. 10.1016/S0140-6736(15)01238-6 26719230

[174] Takayama Y Hattori N Hamada H Masuda T Omori K Akita S Inhibition of PAI-1 limits tumor angiogenesis regardless of angiogenic stimuli in malignant pleural mesothelioma. Cancer Res (2016) 76(11):3285–94. 10.1158/0008-5472.CAN-15-1796 27197170

[175] Comertpay S Pastorino S Tanji M Mezzapelle R Strianese O Napolitano A Evaluation of clonal origin of malignant mesothelioma. J Transl Med (2014) 12:301. 10.1186/s12967-014-0301-3 25471750 PMC4255423

[176] Turgut Cosan D Ak G Dag I Soyocak A Dikmen G Dal A [Drug carrier nanosystems in malignant pleural mesothelioma]. Tuberk Toraks (2016) 64(1):60–8. 27266287

[177] Goricar K Kovac V Dolzan V. Clinical-pharmacogenetic models for personalized cancer treatment: application to malignant mesothelioma. Sci Rep (2017) 7:46537. 10.1038/srep46537 28422153 PMC5396189

[178] Rolfo C Castiglia M Hong D Alessandro R Mertens I Baggerman G Liquid biopsies in lung cancer: the new ambrosia of researchers. Biochim Biophys Acta (2014) 1846(2):539–46. 10.1016/j.bbcan.2014.10.001 25444714

[179] Pass HI Lott D Lonardo F Harbut M Liu Z Tang N Asbestos exposure, pleural mesothelioma, and serum osteopontin levels. N Engl J Med (2005) 353(15):1564–73. 10.1056/NEJMoa051185 16221779

[180] Pass HI Levin SM Harbut MR Melamed J Chiriboga L Donington J Fibulin-3 as a blood and effusion biomarker for pleural mesothelioma. N Engl J Med (2012) 367(15):1417–27. 10.1056/NEJMoa1115050 23050525 PMC3761217

[181] Creaney J Dick IM Meniawy TM Leong SL Leon JS Demelker Y Comparison of fibulin-3 and mesothelin as markers in malignant mesothelioma. Thorax (2014) 69(10):895–902. 10.1136/thoraxjnl-2014-205205 25037982 PMC4174124

[182] Creaney J Dick IM Robinson BW. Discovery of new biomarkers for malignant mesothelioma. Curr Pulmonol Rep (2015) 4(1):15–21. 10.1007/s13665-015-0106-8 25927434 PMC4356891

[183] Pass HI Goparaju C Espin-Garcia O Donington J Carbone M Patel D Plasma biomarker enrichment of clinical prognostic indices in malignant pleural mesothelioma. J Thorac Oncol (2016) 11(6):900–9. 10.1016/j.jtho.2016.02.006 26903362 PMC5978729

[184] Mairinger FD Vollbrecht C Flom E Christoph DC Schmid KW Kollmeier J Folic acid phenotype (FAP) is a superior biomarker predicting response to pemetrexed-based chemotherapy in malignant pleural mesothelioma. Oncotarget (2017) 8(23):37502–10. 10.18632/oncotarget.16398 28415584 PMC5514925

[185] Hassan R Ho M. Mesothelin targeted cancer immunotherapy. Eur J Cancer (2008) 44(1):46–53. 10.1016/j.ejca.2007.08.028 17945478 PMC2265108

[186] Robinson BW Creaney J Lake R Nowak A Musk AW de Klerk N Mesothelin-family proteins and diagnosis of mesothelioma. Lancet (2003) 362(9396):1612–6. 10.1016/S0140-6736(03)14794-0 14630441

[187] Scherpereel A Grigoriu B Conti M Gey T Gregoire M Copin MC Soluble mesothelin-related peptides in the diagnosis of malignant pleural mesothelioma. Am J Respir Crit Care Med (2006) 173(10):1155–60. 10.1164/rccm.200511-1789OC 16456138

[188] Pass HI Wali A Tang N Ivanova A Ivanov S Harbut M Soluble mesothelin-related peptide level elevation in mesothelioma serum and pleural effusions. Ann Thorac Surg (2008) 85(1):265–72; discussion 72. 10.1016/j.athoracsur.2007.07.042 18154821

[189] Lagniau S Lamote K van Meerbeeck JP Vermaelen KY. Biomarkers for early diagnosis of malignant mesothelioma: do we need another moonshot? Oncotarget (2017) 8(32):53751–62. 10.18632/oncotarget.17910 28881848 PMC5581147

[190] Jiang Z Ying S Shen W He X Chen J Xia H Plasma fibulin-3 as a potential biomarker for patients with asbestos-related diseases in the Han population. Dis Markers (2017) 2017:1725354. 10.1155/2017/1725354 29200597 PMC5671709

[191] Grigoriu BD Scherpereel A Devos P Chahine B Letourneux M Lebailly P Utility of osteopontin and serum mesothelin in malignant pleural mesothelioma diagnosis and prognosis assessment. Clin Cancer Res (2007) 13(10):2928–35. 10.1158/1078-0432.CCR-06-2144 17504993

[192] Corallini A Mazzoni E Taronna A Manfrini M Carandina G Guerra G Specific antibodies reacting with simian virus 40 capsid protein mimotopes in serum samples from healthy blood donors. Hum Immunol (2012) 73(5):502–10. 10.1016/j.humimm.2012.02.009 22387152

[193] Ribeiro T Fleury MJ Granieri E Castellazzi M Martini F Mazzoni E Investigation of the prevalence of antibodies against neurotropic polyomaviruses BK, JC and SV40 in sera from patients affected by multiple sclerosis. Neurol Sci (2010) 31(4):517–21. 10.1007/s10072-010-0353-y 20552238

[194] Tognon M Corallini A Manfrini M Taronna A Butel JS Pietrobon S Specific antibodies reacting with SV40 large T antigen mimotopes in serum samples of healthy subjects. PLoS One (2016) 11(1):e0145720. 10.1371/journal.pone.0145720 26731525 PMC4701414

[195] Budhu A Ji J Wang XW The clinical potential of microRNAs. J Hematol Oncol (2010) 3:37 10.1186/1756-8722-3-37 20925959 PMC2958878

[196] Reid G. MicroRNAs in mesothelioma: from tumour suppressors and biomarkers to therapeutic targets. J Thorac Dis (2015) 7(6):1031–40. 10.3978/j.issn.2072-1439.2015.04.56 26150916 PMC4466421

[197] Saito Y Nakaoka T Saito H. MicroRNA-34a as a therapeutic agent against human cancer. J Clin Med (2015) 4(11):1951–9. 10.3390/jcm4111951 26580663 PMC4663478

[198] Kinet V Halkein J Dirkx E Windt LJ. Cardiovascular extracellular microRNAs: emerging diagnostic markers and mechanisms of cell-to-cell RNA communication. Front Genet (2013) 4:214. 10.3389/fgene.2013.00214 24273550 PMC3824095

[199] Valadi H Ekstrom K Bossios A Sjostrand M Lee JJ Lotvall JO. Exosome-mediated transfer of mRNAs and microRNAs is a novel mechanism of genetic exchange between cells. Nat Cell Biol (2007) 9(6):654–9. 10.1038/ncb1596 17486113

[200] Sethi S Kong D Land S Dyson G Sakr WA Sarkar FH. Comprehensive molecular oncogenomic profiling and miRNA analysis of prostate cancer. Am J Transl Res (2013) 5(2):200–11. 23573364 PMC3612515

[201] Qi J Wang J Katayama H Sen S Liu SM. Circulating microRNAs (cmiRNAs) as novel potential biomarkers for hepatocellular carcinoma. Neoplasma (2013) 60(2):135–42. 10.4149/neo_2013_018 23259781 PMC3869230

[202] Volinia S Calin GA Liu CG Ambs S Cimmino A Petrocca F A microRNA expression signature of human solid tumors defines cancer gene targets. Proc Natl Acad Sci U S A (2006) 103(7):2257–61. 10.1073/pnas.0510565103 16461460 PMC1413718

[203] Micolucci L Akhtar MM Olivieri F Rippo MR Procopio AD. Diagnostic value of microRNAs in asbestos exposure and malignant mesothelioma: systematic review and qualitative meta-analysis. Oncotarget (2016) 7(36):58606–37. 10.18632/oncotarget.9686 27259231 PMC5295457

[204] Smith B Agarwal P Bhowmick NA. MicroRNA applications for prostate, ovarian and breast cancer in the era of precision medicine. Endocr Relat Cancer (2017) 24(5):R157–72. 10.1530/ERC-16-0525 28289080 PMC5446589

[205] Shah MY Ferrajoli A Sood AK Lopez-Berestein G Calin GA. MicroRNA therapeutics in cancer – an emerging concept. EBioMedicine (2016) 12:34–42. 10.1016/j.ebiom.2016.09.017 27720213 PMC5078622

[206] Kao SC Fulham M Wong K Cooper W Brahmbhatt H MacDiarmid J A significant metabolic and radiological response after a novel targeted microRNA-based treatment approach in malignant pleural mesothelioma. Am J Respir Crit Care Med (2015) 191(12):1467–9. 10.1164/rccm.201503-0461LE 26075427

[207] Luistro L He W Smith M Packman K Vilenchik M Carvajal D Preclinical profile of a potent gamma-secretase inhibitor targeting notch signaling with in vivo efficacy and pharmacodynamic properties. Cancer Res (2009) 69(19):7672–80. 10.1158/0008-5472.CAN-09-1843 19773430 PMC5260798

[208] Pannuti A Foreman K Rizzo P Osipo C Golde T Osborne B Targeting Notch to target cancer stem cells. Clin Cancer Res (2010) 16(12):3141–52. 10.1158/1078-0432.CCR-09-2823 20530696 PMC3008160

[209] Gu JW Rizzo P Pannuti A Golde T Osborne B Miele L Notch signals in the endothelium and cancer “stem-like” cells: opportunities for cancer therapy. Vasc Cell (2012) 4:7 10.1186/2045-824X-4-7 22487493 PMC3348040

[210] Takebe N Miele L Harris PJ Jeong W Bando H Kahn M Targeting Notch, hedgehog, and Wnt pathways in cancer stem cells: clinical update. Nat Rev Clin Oncol (2015) 12(8):445–64. 10.1038/nrclinonc.2015.61 25850553 PMC4520755

[211] Jia X Wang W Xu Z Wang S Wang T Wang M A humanized anti-DLL4 antibody promotes dysfunctional angiogenesis and inhibits breast tumor growth. Sci Rep (2016) 6:27985. 10.1038/srep27985 27301650 PMC4908374

[212] Li D Masiero M Banham AH Harris AL. The notch ligand JAGGED1 as a target for anti-tumor therapy. Front Oncol (2014) 4:254. 10.3389/fonc.2014.00254 25309874 PMC4174884

[213] Pattarozzi A Carra E Favoni RE Wurth R Marubbi D Filiberti RA The inhibition of FGF receptor 1 activity mediates sorafenib antiproliferative effects in human malignant pleural mesothelioma tumor-initiating cells. Stem Cell Res Ther (2017) 8(1):119. 10.1186/s13287-017-0573-7 28545562 PMC5445511

[214] Uematsu K Seki N Seto T Isoe C Tsukamoto H Mikami I Targeting the Wnt signaling pathway with dishevelled and cisplatin synergistically suppresses mesothelioma cell growth. Anticancer Res (2007) 27(6B):4239–42. 18225596

[215] Woodard GA Yang YL You L Jablons DM. Drug development against the hippo pathway in mesothelioma. Transl Lung Cancer Res (2017) 6(3):335–42. 10.21037/tlcr.2017.06.02 28713678 PMC5504108

[216] Sanalkumar R Dhanesh SB James J. Non-canonical activation of Notch signaling/target genes in vertebrates. Cell Mol Life Sci (2010) 67(17):2957–68. 10.1007/s00018-010-0391-x 20458516 PMC11115867

[217] Lin S Negulescu A Bulusu S Gibert B Delcros JG Ducarouge B Non-canonical NOTCH3 signalling limits tumour angiogenesis. Nat Commun (2017) 8:16074. 10.1038/ncomms16074 28719575 PMC5520050

[218] Lai J Zhou Z Tang XJ Gao ZB Zhou J Chen SQ. A tumor-specific neo-antigen caused by a frameshift mutation in BAP1 is a potential personalized biomarker in malignant peritoneal mesothelioma. Int J Mol Sci (2016) 17(5):E739. 10.3390/ijms17050739 27187383 PMC4881561

[219] Bueno R Stawiski EW Goldstein LD Durinck S De Rienzo A Modrusan Z Comprehensive genomic analysis of malignant pleural mesothelioma identifies recurrent mutations, gene fusions and splicing alterations. Nat Genet (2016) 48(4):407–16. 10.1038/ng.3520 26928227

